# Factor analysis for gene regulatory networks and transcription factor activity profiles

**DOI:** 10.1186/1471-2105-8-61

**Published:** 2007-02-23

**Authors:** Iosifina Pournara, Lorenz Wernisch

**Affiliations:** 1 School of Crystallography, Birkbeck College, University of London, London, UK

## Abstract

**Background:**

Most existing algorithms for the inference of the structure of gene regulatory networks from gene expression data assume that the activity levels of transcription factors (TFs) are proportional to their mRNA levels. This assumption is invalid for most biological systems. However, one might be able to reconstruct unobserved activity profiles of TFs from the expression profiles of target genes. A simple model is a two-layer network with unobserved TF variables in the first layer and observed gene expression variables in the second layer. TFs are connected to regulated genes by weighted edges. The weights, known as *factor loadings*, indicate the strength and direction of regulation. Of particular interest are methods that produce sparse networks, networks with few edges, since it is known that most genes are regulated by only a small number of TFs, and most TFs regulate only a small number of genes.

**Results:**

In this paper, we explore the performance of five factor analysis algorithms, Bayesian as well as classical, on problems with biological context using both simulated and real data. Factor analysis (FA) models are used in order to describe a larger number of observed variables by a smaller number of unobserved variables, the *factors*, whereby all correlation between observed variables is explained by common factors. Bayesian FA methods allow one to infer sparse networks by enforcing sparsity through priors. In contrast, in the classical FA, matrix rotation methods are used to enforce sparsity and thus to increase the interpretability of the inferred factor loadings matrix. However, we also show that Bayesian FA models that do not impose sparsity through the priors can still be used for the reconstruction of a gene regulatory network if applied in conjunction with matrix rotation methods. Finally, we show the added advantage of merging the information derived from all algorithms in order to obtain a combined result.

**Conclusion:**

Most of the algorithms tested are successful in reconstructing the connectivity structure as well as the TF profiles. Moreover, we demonstrate that if the underlying network is sparse it is still possible to reconstruct hidden activity profiles of TFs to some degree without prior connectivity information.

## Background

Factor analysis (FA) as well as principal component analysis (PCA) is used to describe a number of observed variables by a smaller number of unobserved variables. Unlike PCA, FA also includes independent additive measurement errors on the observed variables. FA assumes that the observed variables become uncorrelated given a set of hidden variables called *factors*. It can also be seen as a clustering method where the variables described by the same factors are highly correlated, thus belonging to the same cluster, while the variables depending on different factors are uncorrelated and placed in different clusters.

FA has been successfully used in a number of areas such as computer vision, pattern recognition, economics and more recently in bioinformatics [1-4]. The suitability of FA for gene expression analysis is also the motivation of this work. Genes are transcribed into mRNAs which in turn are translated into proteins. Some of these proteins activate or inhibit, as transcription factors (TFs), the transcription of a number of other genes creating a complex *gene regulatory network*. The number of transcription factors is much smaller than the number of transcribed genes and most genes are regulated only by a small number of transcription factors. Hence, the matrix that describes the connections between the transcription factors and the regulated genes is sparse. Using microarrays, mRNA levels of thousands of genes can be measured simultaneously, but no direct information is obtained about TF activity. Our aim is two-fold: to identify the genes regulated by a common TF, that is, to reconstruct the connectivity structure and weights in a two-layer network, and to reconstruct the activity profile of each TF.

Liao et al. [5] have suggested the use of a network component analysis (NCA) algorithm for reconstructing the profiles of the TFs (see also [6] and [7]), while Boulesteix and Strimmer [8] have used an approach based on partial least squares regression. They have both shown that such methods can faithfully reconstruct the expression profiles of the TFs. However, both methods rely heavily on the availability of connectivity information. Nonzero positions in the *factor loadings matrix*, which describes the connections between the factors and the genes, need to be specified in advance. The algorithms then estimate the values at these positions (which might turn out to be zero). This is a strong limitation since often only little information about genes regulated by specific TFs is available. FA models are faced with a much harder task where both the structure of the factor loadings matrix and the activity profiles of the factors have to be reconstructed. Independent component analysis (ICA) has also been widely used in bioinformatics (see for example [9,10] and [11]). This approach assumes that the transcription factors are statistically independent. A comparison of NCA and ICA can be found in Liao et al. [5], and thus ICA will not be considered further here. A further advantage of the Bayesian FA models is that any information about the underlying structure can be easily incorporated through priors. This improves performance, but is not required for the algorithms to be applicable in the first place, as in the case of NCA and its generalisations.

Hinton et al. [12] first introduced an EM algorithm for factor analysis in order to model the manifolds of digitised images of handwritten digits. Later Ghahramani and Hinton [13] presented an exact EM algorithm for both factor analyzers and mixtures of factor analyzers. More recently, Utsugi and Kumagai [14] used a Gibbs sampler instead of the EM algorithm suggested by Ghahramani and Hinton [13] for mixtures of factor analyzers. West [3] was the first to introduce Bayesian factor analysis in the bioinformatics field. To accommodate the required sparsity regarding the connections between the factors and the genes, he suggested the use of a mixture prior on the factor loadings matrix. As is shown in the results section, the predicted factor loadings matrix has the desired sparsity, at the expense of increasing computing time as the number of hidden variables increases. Recently, Sabatti and James [4] have used the framework by West [3] for the reconstruction of transcription factor profiles. In order to avoid the computational burden of estimating the factor loadings matrix at each step of the Gibbs sampler and to facilitate the reconstruction process, they set a large number of entries to zero based on information obtained from the Vocabulon algorithm [15]. This algorithm scans DNA sequences for multiple motifs and associates with each transcription factor a probability of binding to a specific site. This approach resembles the approach of Liao et al. [5], and Boulesteix and Strimmer [8] where the structure of the factor loadings matrix is given in advance.

Note that the algorithms of Ghahramani and Hinton [13], and Utsugi and Kumagai [14] have not previously been applied to biological data, and that the algorithm of Sabatti and James [4] is an adaptation of the algorithm of West [3] with the difference that an informative prior is used for the factor loadings matrix. Also, Sabatti and James [4] applied the FA model to yeast and *E. coli *data, while West [3] applied his algorithm to cancer data.

In this paper, we suggest the use of Fokoue's algorithm [16] as an alternative to West's algorithm [3]. This algorithm utilises a Gamma prior distribution on the variance of the factor loadings matrix that imposes the required sparsity but, at the same time, avoids the computational burden introduced by the use of a mixture prior [3]. Since this algorithm avoids the combinatorial problem of West's algorithm, a prior knowledge on the underlying model is not required. At the same time, we give a thorough review of all FA algorithms mentioned above and examine the applicability of those algorithms to biological data. To the best of our knowledge such a comparison of FA algorithms in the scope of analyzing microarray data has not been presented before. Moreover, we extend these algorithms by suggesting a further factor rotation analysis which produces additional sparsity of the factor loadings matrix. This additional sparsity not only facilitates the interpretation of the results, but it is also useful in a biological context where a very sparse matrix is required. Finally, we show that merging the information provided by each algorithm to obtain a combined result leads to better performance. The algorithms are compared based on their ability to reconstruct the underlying factor loadings matrix and the profiles of the transcription factors.

The comparison is done on both simulated data where the true answer is known and on experimental data. We evaluate the performance of the algorithms on the Hemoglobin data obtained by Liao et al. [5] and on the Escherichia coli (*E. coli*) data in Kao et al. [6]. Although time series data show correlation that is ignored in a factor analysis, which in fact assumes independence across data points, we used these data sets for comparison of our results with that in Liao et al. [5], Kao et al. [6], and Boulesteix and Strimmer [8].

### Factor analysis model

Let us assume that we have a random observed vector variable *x *of *P *dimensions, *x *= (*x*_1_,..., *x*_*P*_)'. We denote an instance of this vector with a superscript *n *and we assume that we have *N *such instances, *x*^*n *^where *n *= 1,..., *N*. Similarly, *f *= (*f*_1_,..., *f*_*K*_)' is a vector of *K *hidden variables, known as *factors*. Note that the number *K *of factors is always smaller than or equal to the number *P *of observed variables. The factor analysis model states that the observed variables are a linear combination of the factors plus a mean and an error term. For case *n*

xn(P × 1)=μ(P × 1)+Λ(P × K)fn(K × 1)+εn(P × 1)     (1)
 MathType@MTEF@5@5@+=feaafiart1ev1aaatCvAUfKttLearuWrP9MDH5MBPbIqV92AaeXatLxBI9gBaebbnrfifHhDYfgasaacH8akY=wiFfYdH8Gipec8Eeeu0xXdbba9frFj0=OqFfea0dXdd9vqai=hGuQ8kuc9pgc9s8qqaq=dirpe0xb9q8qiLsFr0=vr0=vr0dc8meaabaqaciaacaGaaeqabaqabeGadaaakeaafaqaaeqaiaaaaaqaamaaxababaGaemiEaG3aaWbaaSqabeaacqWGUbGBaaaabaGaeiikaGIaemiuaaLaeeiiaaIaey41aqRaeeiiaaIaeGymaeJaeiykaKcabeaaaOqaaiabg2da9aqaamaaxababaacciGae8hVd0galeaacqGGOaakcqWGqbaucqqGGaaicqGHxdaTcqqGGaaicqaIXaqmcqGGPaqkaeqaaaGcbaGaey4kaScabaWaaCbeaeaacqqHBoataSqaaiabcIcaOiabdcfaqjabbccaGiabgEna0kabbccaGiabdUealjabcMcaPaqabaaakeaadaWfqaqaaiabdAgaMnaaCaaaleqabaGaemOBa4gaaaqaaiabcIcaOiabdUealjabbccaGiabgEna0kabbccaGiabigdaXiabcMcaPaqabaaakeaacqGHRaWkaeaadaWfqaqaaiab=v7aLnaaCaaaleqabaGaemOBa4gaaaqaaiabcIcaOiabdcfaqjabbccaGiabgEna0kabbccaGiabigdaXiabcMcaPaqabaaaaOGaaCzcaiaaxMaadaqadaqaaiabigdaXaGaayjkaiaawMcaaaaa@6626@

where *μ *= (*μ*_1_,..., *μ*_*P*_)' and *ε*^*n *^= (ε1n
 MathType@MTEF@5@5@+=feaafiart1ev1aaatCvAUfKttLearuWrP9MDH5MBPbIqV92AaeXatLxBI9gBaebbnrfifHhDYfgasaacH8akY=wiFfYdH8Gipec8Eeeu0xXdbba9frFj0=OqFfea0dXdd9vqai=hGuQ8kuc9pgc9s8qqaq=dirpe0xb9q8qiLsFr0=vr0=vr0dc8meaabaqaciaacaGaaeqabaqabeGadaaakeaaiiGacqWF1oqzdaqhaaWcbaGaeGymaedabaGaemOBa4gaaaaa@30DC@,...,εPn
 MathType@MTEF@5@5@+=feaafiart1ev1aaatCvAUfKttLearuWrP9MDH5MBPbIqV92AaeXatLxBI9gBaebbnrfifHhDYfgasaacH8akY=wiFfYdH8Gipec8Eeeu0xXdbba9frFj0=OqFfea0dXdd9vqai=hGuQ8kuc9pgc9s8qqaq=dirpe0xb9q8qiLsFr0=vr0=vr0dc8meaabaqaciaacaGaaeqabaqabeGadaaakeaaiiGacqWF1oqzdaqhaaWcbaGaemiuaafabaGaemOBa4gaaaaa@3115@)' are column vectors of dimension *P *with elements corresponding to the mean and the error of the *P *observed variables. The vector *μ *is the same for all cases. Λ is the unobserved *transition matrix *also referred to as the *factor loadings matrix*. The factor loadings matrix has *P *× *K *dimensions. That is, each column corresponds to a factor and each row corresponds to an observed variable. The entries of the factor loadings matrix indicate the strength of the dependence of each observed variable on each factor. For example, if *λ*_*pk *_is zero, then variable *x*_*p *_is independent of factor *f*_*k*_. In matrix form equation 1 is

X(P × N)=M(P × N)+Λ(P × K)F(K × N)+E(P × N)     (2)
 MathType@MTEF@5@5@+=feaafiart1ev1aaatCvAUfKttLearuWrP9MDH5MBPbIqV92AaeXatLxBI9gBaebbnrfifHhDYfgasaacH8akY=wiFfYdH8Gipec8Eeeu0xXdbba9frFj0=OqFfea0dXdd9vqai=hGuQ8kuc9pgc9s8qqaq=dirpe0xb9q8qiLsFr0=vr0=vr0dc8meaabaqaciaacaGaaeqabaqabeGadaaakeaafaqaaeqaiaaaaaqaamaaxababaGaemiwaGfaleaacqGGOaakcqWGqbaucqqGGaaicqGHxdaTcqqGGaaicqWGobGtcqGGPaqkaeqaaaGcbaGaeyypa0dabaWaaCbeaeaacqWGnbqtaSqaaiabcIcaOiabdcfaqjabbccaGiabgEna0kabbccaGiabd6eaojabcMcaPaqabaaakeaacqGHRaWkaeaadaWfqaqaaiabfU5ambWcbaGaeiikaGIaemiuaaLaeeiiaaIaey41aqRaeeiiaaIaem4saSKaeiykaKcabeaaaOqaamaaxababaGaemOrayealeaacqGGOaakcqWGlbWscqqGGaaicqGHxdaTcqqGGaaicqWGobGtcqGGPaqkaeqaaaGcbaGaey4kaScabaWaaCbeaeaacqWGfbqraSqaaiabcIcaOiabdcfaqjabbccaGiabgEna0kabbccaGiabd6eaojabcMcaPaqabaaaaOGaaCzcaiaaxMaadaqadaqaaiabikdaYaGaayjkaiaawMcaaaaa@60BE@

where *X *= (*x*^1^,..., *x*^*N*^), *F *= (*f*^1^,..., *f*^*N*^), *E *= (*ε*^1^,..., *ε*^*N*^), *M *= *μe*_*N *_with *e*_*N *_an *N *dimensional row vector of ones. FA models assume that the error terms *ε*^*n *^are independent, and multivariate normally distributed with mean zero and covariance matrix Ψ, *ε*^*n *^~ N
 MathType@MTEF@5@5@+=feaafiart1ev1aaatCvAUfKttLearuWrP9MDH5MBPbIqV92AaeXatLxBI9gBaebbnrfifHhDYfgasaacH8akY=wiFfYdH8Gipec8Eeeu0xXdbba9frFj0=OqFfea0dXdd9vqai=hGuQ8kuc9pgc9s8qqaq=dirpe0xb9q8qiLsFr0=vr0=vr0dc8meaabaqaciaacaGaaeqabaqabeGadaaakeaat0uy0HwzTfgDPnwy1egaryqtHrhAL1wy0L2yHvdaiqaacqWFneVtaaa@383A@(0, Ψ), where Ψ = diag(ψ12
 MathType@MTEF@5@5@+=feaafiart1ev1aaatCvAUfKttLearuWrP9MDH5MBPbIqV92AaeXatLxBI9gBaebbnrfifHhDYfgasaacH8akY=wiFfYdH8Gipec8Eeeu0xXdbba9frFj0=OqFfea0dXdd9vqai=hGuQ8kuc9pgc9s8qqaq=dirpe0xb9q8qiLsFr0=vr0=vr0dc8meaabaqaciaacaGaaeqabaqabeGadaaakeaaiiGacqWFipqEdaqhaaWcbaGaeGymaedabaGaeGOmaidaaaaa@3090@,..., ψP2
 MathType@MTEF@5@5@+=feaafiart1ev1aaatCvAUfKttLearuWrP9MDH5MBPbIqV92AaeXatLxBI9gBaebbnrfifHhDYfgasaacH8akY=wiFfYdH8Gipec8Eeeu0xXdbba9frFj0=OqFfea0dXdd9vqai=hGuQ8kuc9pgc9s8qqaq=dirpe0xb9q8qiLsFr0=vr0=vr0dc8meaabaqaciaacaGaaeqabaqabeGadaaakeaaiiGacqWFipqEdaqhaaWcbaGaemiuaafabaGaeGOmaidaaaaa@30C9@). Thus the probability distribution of *x *for each observed case *n *has a multivariate normal density given by

p(xn|fn,Λ,μ,Ψ)=N(xn|μ+Λfn,Ψ)=(2π)−P/2|Ψ|−1/2×exp⁡(−12(xn−μ−Λfn)′Ψ−1(xn−μ−Λfn))     (3)
 MathType@MTEF@5@5@+=feaafiart1ev1aaatCvAUfKttLearuWrP9MDH5MBPbIqV92AaeXatLxBI9gBaebbnrfifHhDYfgasaacH8akY=wiFfYdH8Gipec8Eeeu0xXdbba9frFj0=OqFfea0dXdd9vqai=hGuQ8kuc9pgc9s8qqaq=dirpe0xb9q8qiLsFr0=vr0=vr0dc8meaabaqaciaacaGaaeqabaqabeGadaaakeaafaqaaeGadaaabaGaemiCaaNaeiikaGIaemiEaG3aaWbaaSqabeaacqWGUbGBaaGccqGG8baFcqWGMbGzdaahaaWcbeqaaiabd6gaUbaakiabcYcaSiabfU5amjabcYcaSGGaciab=X7aTjabcYcaSiabfI6azjabcMcaPaqaaiabg2da9aqaamrtHrhAL1wy0L2yHvtyaeHbnfgDOvwBHrxAJfwnaGabaiab+1q8ojabcIcaOiabdIha4naaCaaaleqabaGaemOBa4gaaOGaeiiFaWNae8hVd0Maey4kaSIaeu4MdWKaemOzay2aaWbaaSqabeaacqWGUbGBaaGccqGGSaalcqqHOoqwcqGGPaqkaeaaaeaacqGH9aqpaeaacqGGOaakcqaIYaGmcqWFapaCcqGGPaqkdaahaaWcbeqaaiabgkHiTiabdcfaqjabc+caViabikdaYaaakmaaemaabaGaeuiQdKfacaGLhWUaayjcSdWaaWbaaSqabeaacqGHsislcqaIXaqmcqGGVaWlcqaIYaGmaaGccqGHxdaTcyGGLbqzcqGG4baEcqGGWbaCdaqadaqaaiabgkHiTmaalaaabaGaeGymaedabaGaeGOmaidaaiabcIcaOiabdIha4naaCaaaleqabaGaemOBa4gaaOGaeyOeI0Iae8hVd0MaeyOeI0Iaeu4MdWKaemOzay2aaWbaaSqabeaacqWGUbGBaaGccuGGPaqkgaqbaiabfI6aznaaCaaaleqabaGaeyOeI0IaeGymaedaaOGaeiikaGIaemiEaG3aaWbaaSqabeaacqWGUbGBaaGccqGHsislcqWF8oqBcqGHsislcqqHBoatcqWGMbGzdaahaaWcbeqaaiabd6gaUbaakiabcMcaPaGaayjkaiaawMcaaaaacaWLjaGaaCzcamaabmaabaGaeG4mamdacaGLOaGaayzkaaaaaa@98B9@

or in matrix notation

p(X|F,Λ,μ,Ψ)=N(X|M+ΛF,Ψ)=(2π)−N/2|Ψ|−1/2×exp⁡(−12tr[(X−M−ΛF)′Ψ−1(X−M−ΛF)])     (4)
 MathType@MTEF@5@5@+=feaafiart1ev1aaatCvAUfKttLearuWrP9MDH5MBPbIqV92AaeXatLxBI9gBamrtHrhAL1wy0L2yHvtyaeHbnfgDOvwBHrxAJfwnaebbnrfifHhDYfgasaacH8akY=wiFfYdH8Gipec8Eeeu0xXdbba9frFj0=OqFfea0dXdd9vqai=hGuQ8kuc9pgc9s8qqaq=dirpe0xb9q8qiLsFr0=vr0=vr0dc8meaabaqaciaacaGaaeqabaWaaeGaeaaakeaafaqaaeGadaaabaGaemiCaaNaeiikaGIaemiwaGLaeiiFaWNaemOrayKaeiilaWIaeu4MdWKaeiilaWccciGae8hVd0MaeiilaWIaeuiQdKLaeiykaKcabaGaeyypa0dabaacdaGae4xdX7KaeiikaGIaemiwaGLaeiiFaWNaemyta0Kaey4kaSIaeu4MdWKaemOrayKaeiilaWIaeuiQdKLaeiykaKcabaaabaGaeyypa0dabaGaeiikaGIaeGOmaiJae8hWdaNaeiykaKYaaWbaaSqabeaacqGHsislcqWGobGtcqGGVaWlcqaIYaGmaaGcdaabdaqaaiabfI6azbGaay5bSlaawIa7amaaCaaaleqabaGaeyOeI0IaeGymaeJaei4la8IaeGOmaidaaOGaey41aqRagiyzauMaeiiEaGNaeiiCaa3aaeWaaeaacqGHsisldaWcaaqaaiabigdaXaqaaiabikdaYaaacqqG0baDcqqGYbGCcqGGBbWwcqGGOaakcqWGybawcqGHsislcqWGnbqtcqGHsislcqqHBoatcqWGgbGrcuGGPaqkgaqbaiabfI6aznaaCaaaleqabaGaeyOeI0IaeGymaedaaOGaeiikaGIaemiwaGLaeyOeI0Iaemyta0KaeyOeI0Iaeu4MdWKaemOrayKaeiykaKIaeiyxa0facaGLOaGaayzkaaaaaiaaxMaacaWLjaWaaeWaaeaacqaI0aanaiaawIcacaGLPaaaaaa@8D88@

where tr is the trace, the sum of the diagonal elements. In the methods section, we discuss in detail the prior and posterior probabilities of the parameters *F*, *μ*, Λ and Ψ, as well as algorithms for their estimation.

### Identifiability problems

As shown in equation 5 in the methods section, the complete density of the data, when factors are integrated out, is given by a normal distribution with covariance matrix Λ*Σ*_*f *_Λ' + Ψ. There is a scale identifiability problem associated with Λ and Σ_*f*_. In order to avoid this problem, we could either restrict the columns of Λ to unit vectors or set Σ_*f *_to the identity matrix. The second approach is often preferred in factor analysis.

There is also an identifiability problem associated with equation 2. Let us assume that we have an orthogonal matrix *Q *of dimensions *K *× *K *with *QQ*' = *Q'Q *= *I*_*K*_. Then we can have

Λ*F *= Λ*QQ*'*F *= Λ*F*

with cov(F*) = cov(*F*). That is, it is not possible to distinguish between Λ and all its possible orthogonal transformations Λ* based on knowledge of the product Λ*F *only. However, as we show in the results section, if the loadings matrix underlying the data generating process is sparse enough, it can often be reconstructed. This can be done either by using sparsity priors on the entries of the loadings matrix in a Bayesian setting or by orthogonal rotations enforcing sparsity (see methods section).

Note that orthogonal transformations also include permutations of the factors. Factors could be ordered by the amount of variance explained. Or, as in the case of regulatory networks, we would have to map known TFs to the inferred factors. In Sabatti and James [4], the factors are constrained by assigning a priori zero values to the factor loadings matrix. Here, we map the TFs to the inferred factors based on previous knowledge about their activity profiles, as for example reported in Kao et al. [6].

## Results and Discussion

We compare the algorithms by Ghahramani and Hinton [13] (Z), Utsugi and Kumagai [14] (U), Fokoue [16] (F), and West [3] (W) on simulated and real biological data. Algorithm W is based on updating hidden indicator variables representing network connections. For a full exploration of the posterior probability, all possible combinations of hidden values need to be evaluated, thus an exponential number of combinations of these variables. We therefore suggest and test a version (Ws) of the algorithm with independent updates of hidden variables. We also compare these Bayesian FA algorithms with classical FA (as implemented in the Matlab function *factoran *(M)).

In order to evaluate the strengths and weakness of such algorithms we simulate comparatively 'easy' data (that is from linear models) to be able to focus on the question how far sparsity in the connectivity allows identification of the loadings and the factor matrix. Moreover, as shown in the PhD thesis by Pournara [17] the assumption of linearity is not a severe one given the small amount of data and the significant amounts of noise present in microarray data, especially after taking logarithms of mRNA abundance levels or ratios (see also Kao et al. [6]). In a second step, instead of resorting to simulated nonlinear data, which would have invited questions about the choice of particular nonlinear functional forms, we apply the algorithms to real microarray data and evaluate their performance there directly.

### Simulated networks

We test the algorithms on simulated networks. For the generation of random networks we start with a description of network characteristics such as the indegree distribution of genes and outdegree distributions of TFs, which we take from known regulatory networks of *E. coli*. For each TF, we then select random genes subject to these constraints. The activity levels of the factors *F *are drawn from a Gaussian distribution with zero mean and covariance matrix *I*. The vector *μ *of means is set to zero. All non-zero loadings are set to 1. A noise term *E*_*p *_is added in each dimension *p *with zero mean and variance ψp2
 MathType@MTEF@5@5@+=feaafiart1ev1aaatCvAUfKttLearuWrP9MDH5MBPbIqV92AaeXatLxBI9gBaebbnrfifHhDYfgasaacH8akY=wiFfYdH8Gipec8Eeeu0xXdbba9frFj0=OqFfea0dXdd9vqai=hGuQ8kuc9pgc9s8qqaq=dirpe0xb9q8qiLsFr0=vr0=vr0dc8meaabaqaciaacaGaaeqabaqabeGadaaakeaaiiGacqWFipqEdaqhaaWcbaGaemiCaahabaGaeGOmaidaaaaa@3109@ as

ψP2=σp2snr
 MathType@MTEF@5@5@+=feaafiart1ev1aaatCvAUfKttLearuWrP9MDH5MBPbIqV92AaeXatLxBI9gBaebbnrfifHhDYfgasaacH8akY=wiFfYdH8Gipec8Eeeu0xXdbba9frFj0=OqFfea0dXdd9vqai=hGuQ8kuc9pgc9s8qqaq=dirpe0xb9q8qiLsFr0=vr0=vr0dc8meaabaqaciaacaGaaeqabaqabeGadaaakeaaiiGacqWFipqEdaqhaaWcbaGaemiuaafabaGaeGOmaidaaOGaeyypa0ZaaSaaaeaacqWFdpWCdaqhaaWcbaGaemiCaahabaGaeGOmaidaaaGcbaacbaGae43CamNae4NBa4Mae4NCaihaaaaa@3A74@

where σp2
 MathType@MTEF@5@5@+=feaafiart1ev1aaatCvAUfKttLearuWrP9MDH5MBPbIqV92AaeXatLxBI9gBaebbnrfifHhDYfgasaacH8akY=wiFfYdH8Gipec8Eeeu0xXdbba9frFj0=OqFfea0dXdd9vqai=hGuQ8kuc9pgc9s8qqaq=dirpe0xb9q8qiLsFr0=vr0=vr0dc8meaabaqaciaacaGaaeqabaqabeGadaaakeaaiiGacqWFdpWCdaqhaaWcbaGaemiCaahabaGaeGOmaidaaaaa@30FE@ is the variance of the data in dimension *p*, and *snr *is a signal to noise ratio. We evaluate the performance of the algorithms by calculating the mean of squared error (MSE) for the predicted factor loadings matrix Λ and the factor matrix *F*. We identify the labels of the factors by choosing the column permutation of *F *that gives the smallest MSE.

As discussed above, the loadings and factor matrices are only identifiable up to a rotation. Sparsity of the true loadings matrix helps to overcome this lack in identifiability. In algorithms F and W the parameters are estimated by imposing sparsity on the loadings matrix directly. Others, not imposing any prior sparsity, cannot be expected to find the correct solution without further processing, for example, by orthogonal transformations to a sparse form. Results can be improved by normalising the column vectors of the loadings matrix before the transformation, that is, by dividing each vector by its Euclidean length. The inverse of the orthogonal transformation of the loadings matrix is used to transform the factor matrix correspondingly. Finally, in order to assess how successful a factor analysis is independently of the identifiability problem for orthogonal transformations, we apply a procrustes orthogonal transformation (that is, one minimising squared vector distances, see methods section) of the column vectors of the reconstructed loadings matrix onto the column vectors of the true loadings matrix. Such rotation is possible since in the case of simulated data the true loadings matrix is known.

#### Simulated E. coli networks

**Figure 1:**
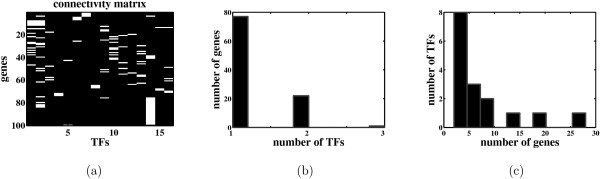
**Factor loadings matrix of the E. coli network**. (a) connectivity matrix of *E. coli *as suggested by Kao et al. [6] (a black entry corresponds to a non interaction while a white entry corresponds to an interaction), (b) distribution of the number of genes regulated by each TF, and (c) distribution of the number of TFs regulating each gene in the *E. coli *network of (a).

We assume that there are only a few TFs in *E. coli *that control the expression profiles of most genes. This assumption is also supported by the connectivity matrix as inferred from RegulonDB [18] and the current literature in Kao et al. [6]. The matrix is reproduced in Figure 1(a). It is very sparse with most genes regulated by 1 to 3 TFs, and with only a few TFs regulating a larger number of genes as shown in Figures 1(b) and 1(c).

**Figure 2:**
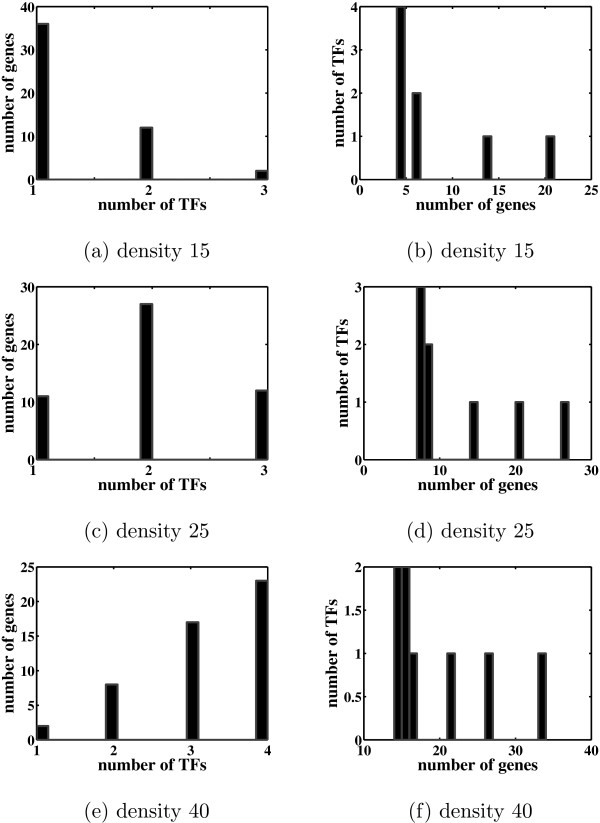
**Distributions of genes and TFs for the simulated networks**. The plots on the left hand side show the distribution of the number of genes regulated by each TF for three networks with densities 15, 25 and 40, respectively. The right hand side plots show the distribution of the number of TFs regulating each gene for the same networks.

We generated random networks consisting of 50 genes and 8 TFs. Since the performance of the algorithms depends on the number of nonzero entries in the loadings matrix Λ, we generated networks with densities ranging from 15 to 40 percent of nonzero entries. Figure 2 shows the distributions of the genes and TFs for three networks with densities of 15, 25 and 40 percent. Networks with density less than 25 have distributions similar to that in the *E. coli *network of Figure 1.

**Figure 3:**
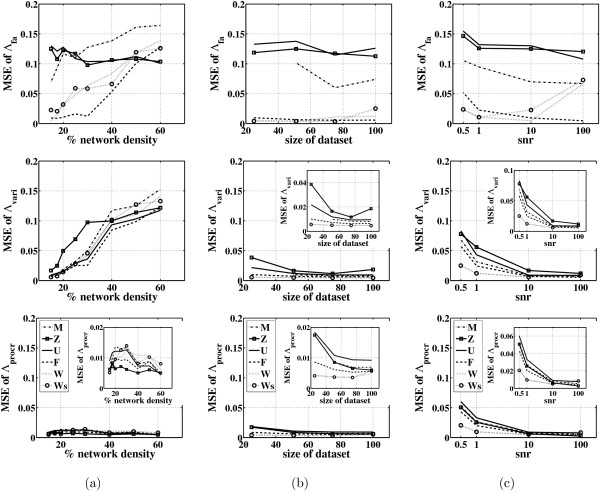
**Evaluation of the FA algorithms on E. coli simulated networks**. Mean squared errors (MSEs) for Λ, the varimax rotated Λ_*vari*_, and the procrustes rotated Λ_*procr *_are shown. The first column (a) shows the MSEs of Λ versus the network density, the second column (b) shows the MSEs of Λ versus the dataset size, and the third column (c) shows the MSEs of Λ for different values of the *snr*. These tests are for networks consisting of 50 genes and 8 TFs. Shown are the mean for 3 different networks. For the definition of the symbols M, Z, U, F, W and Ws see page 6.

Figure 3(a) shows the MSE for the Λ matrix for all the FA algorithms and for different network densities. Shown are the mean value for three random networks for each density. From each network 100 data points were generated, and the *snr *was set to 10. For sparse networks algorithms W and F give a smaller MSE than the other algorithms. However, both algorithms perform worse than algorithms Z and U on dense networks. The sparsity priors in W and F obviously hamper reconstruction of dense networks. Classical FA shows an average performance for sparse networks but decreasing performance for dense ones. Algorithm W performs better than algorithm F only for extremely sparse networks. The version Ws of algorithm W with independent updates of entries in Λ gives results similar to that of W which uses a block update, but with a much faster Gibbs sampling step.

Figure 3(a) also shows the MSE for the varimax and procrustes rotated matrix Λ_*rot*_. Varimax and quartimax rotation give similar results. The equamax rotation gives a slightly higher MSE for sparse matrices and lower MSE for dense matrices (results not shown). Once a varimax rotation is applied to matrices obtained by the FA algorithms, the difference between them regarding the MSE is significantly reduced. It appears that the performance of algorithm F for sparse matrices is better without a varimax rotation; actually so much so that algorithm F is still better than all the other algorithms even after application of varimax. The procrustes rotation indicates the ability of all the FA algorithms to reconstruct a factor loadings matrix that has a very small MSE. However, it also shows that finding the best possible rotation is difficult.

Two more tests were performed to investigate the behavior of the FA algorithms on datasets of different size (ranging from 25 to 100 cases) and data generated with different values of *snr *(ranging from 0.5 to 100). Note that the classical FA algorithm uses the covariance matrix of the data and thus the number of cases must be greater than the number of variables. That is, the *factoran *script was not run for datasets of 25 cases. These two tests were applied to networks with density 15. Figure 3(b) shows again that algorithms Z and U perform similarly regardless of the number of cases in the dataset. Moreover, algorithms F and W also perform similarly and have a much smaller MSE than the other algorithms. Once the varimax rotation is applied, all the algorithms give a similar performance with a smaller MSE achieved as the number of cases increases. For sparse networks with small densities even a very small dataset is enough to reconstruct the factor loadings matrix. The procrustes rotation indicates that algorithm W produces a factor loadings matrix which, if properly rotated, is very close to the true matrix for very sparse networks.

Figure 3(c) shows the results for different values of *snr*. As the amount of noise increases, the performance of most algorithms decreases. Algorithm F has the best performance overall. Varimax rotation improves the performances of the other algorithms and makes them comparable to the results of F and W. Note that algorithm W seems to perform worse when the data are free of noise (snr 100) than when there is at least some small amount of noise (snr 10). However, when we apply varimax rotation to this algorithm we see that the performance decreases indeed with increasing amounts of noise.

**Figure 4:**
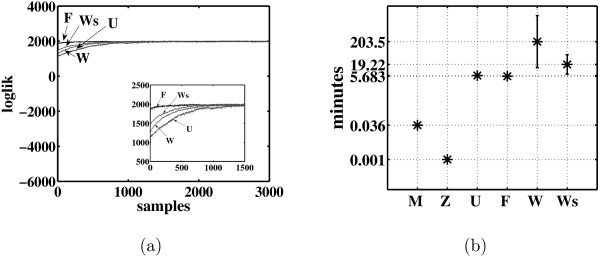
**Convergence test and processing time**. (a) convergence test for the Gibbs sampling algorithms, and (b) the average time consumed by each algorithm.

Figure 4(a) shows the change in the log likelihood for a chosen representative run over 3000 cycles of the Gibbs sampling for algorithms U, F and W. It suggests that all algorithms converge, but algorithm F converges faster than the others. Finally, Figure 4(b) shows the average time consumed by each algorithm. The number of burn-in and sample collection steps (3000) is the same for all the FA algorithms. As mentioned above, for very sparse networks algorithm W produces a better result than algorithms Z, U and the classical FA, but it requires considerably longer time for convergence when the number of factors and genes is large. The results of our version of algorithm W with single updates (Ws) and algorithm W are similar, while Ws is approximately 10 times faster than W. Note that the EM algorithm Z and classical FA are the fastest FA algorithms by reaching convergence within a few seconds. Algorithm Z was downloaded from [19]. All the other FA algorithms were also implemented in MATLAB and run on a 3.06 Ghz Xeon cluster.

Summarising, algorithms F and W perform better on sparse matrices than algorithms Z, U and M because they implicitly capture the required sparsity on the factor loadings matrix. However, if an appropriate orthogonal rotation of the matrices Λ and *F *is applied, the performances of all the FA algorithms are enhanced and become comparable.

### Biological data

We further compare the FA algorithms to two biological datasets; the Hemoglobin dataset from Liao et al. [5], where the connectivity matrix and the profiles of the factors are known to some degree, and the *E. coli *dataset, where the TF profiles and some interactions have been suggested by Kao et al. [6].

#### Hemoglobin dataset

The absorbance spectra of seven hemoglobin solutions (*M *1,..., *M *7) were measured in Liao et al. [5]. Each spectrum is the outcome of a linear combination of the concentrations of three components: oxyhemoglobin (OxyHb), methemoglobin (MetHb) and cyano-methemoglobin (CyanoHb). This dataset consists of 321 measurements for each of the seven hemoglobin solutions.

We first compared the algorithms by Fokoue [16], West [3], and by Tran et al. [7] (GNCA) fixing the positions of zeros in the loadings matrix. Note that the algorithm by Tran et al. [7] requires this connectivity matrix as an input and is unlikely to work properly without this information. Tran et al. [7] have presented an extension of the NCA algorithm [5], the GNCA (generalised network component analysis) algorithm. For details regarding the different versions of the GNCA algorithm see [7]. We present the results for versions GNCA and GNCA_*r*_. Each algorithm was run 20 times. For algorithms GNCA and GNCA_*r*_, we consider the run with the least MSE, while for the FA algorithms we consider the average of these runs.

**Figure 5:**
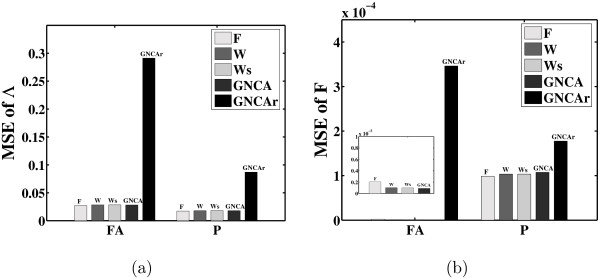
**Reconstruction of the factor loadings matrix for the Hemoglobin data**. Mean square errors (MSEs) for (a) the factor loadings matrix Λ and (b) the factors matrix *F*. The positions of the zero entries in the loadings matrix are given a priori. FA stands for the output of a given FA algorithm. The procrustes (P) factor rotation method is applied to this output to indicate the performance of the algorithms when the best possible rotation is achieved.

**Figure 6:**
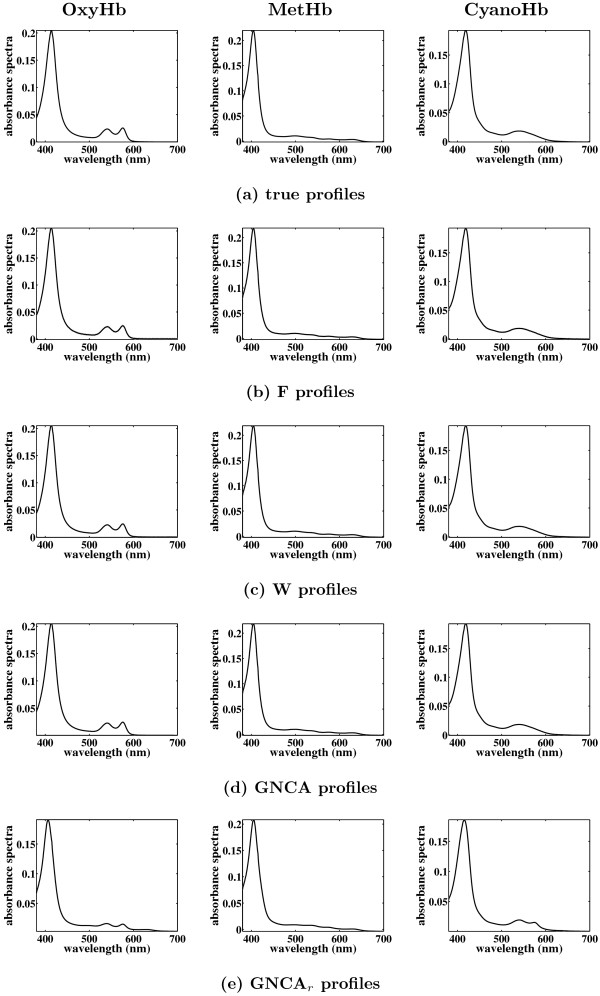
**Reconstruction of the factors matrix for the Hemoglobin data**. Shown are (a) the true profiles of OxyHb, MetHb and CyanoHb, (b) the reconstructed profiles given by algorithm F, (c) the reconstructed profiles given by algorithm W, (d) the reconstructed profiles given by algorithm GNCA, and (e) the reconstructed profiles given by algorithm GNCA_*r*_. The positions of the zero entries in the loadings matrix are given a priori. The light gray curves are the profiles given by the 20 different Gibbs sampling runs, and the black curves are the average profiles. In these figures, the average profile of each factor coincides with its profile given by each single run.

As shown in Figure 5(a), the MSE in the estimation of Λ is approximately equal for all algorithms except GNCA_*r*_, and it is very similar before and after procrustes rotation. This figure indicates that fixing the zero loadings simplifies the task of identifying the underlying factor loadings matrix considerably. Figure 5(b) shows the MSE in the estimation of the factor profiles, and these profiles are plotted in Figure 6. The MSE for the reconstruction of the factor profiles is close to zero for all the algorithms except the algorithm GNCA_*r*_. We used the inverse of the rotation matrix returned for Λ by the procrustes method to rotate the factors. The rotation increases the MSE of the factors since the best rotation for Λ is not necessarily the best rotation for *F*. However, it is still considerably small.

**Figure 7:**
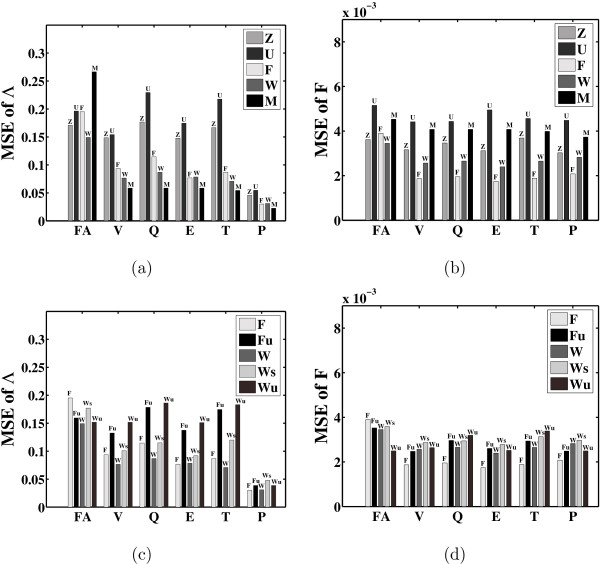
**Reconstruction of the factor loadings matrix for the Hemoglobin data**. Mean square errors (MSEs) for (a) and (c) the factor loadings matrix Λ, and (b) and (d) the factors matrix *F*. The positions of the zero entries in the loadings matrix are not given a priori. FA stands for the output of a given FA algorithm. On this output, a number of factor rotation methods (varimax (V), quartimax (Q), equamax (E), tanh (T) and procrustes (P)) are evaluated based on the MSE. (c) and (d) show the performance of algorithms F and W under different priors regarding the loadings matrix (for further details see section *Hemoglobin dataset*).

We also evaluated the algorithms without providing prior information about the underlying structure of the factor loadings matrix. This can, of course, only be done for the FA algorithms. Figure 7(a) shows the MSE of Λ as given by each algorithm. It also shows the MSE after performing varimax, quartimax, equamax, tanh, and procrustes rotation. Most FA algorithms perform equally well in predicting the values of the loadings of Λ. This is probably due to the fact that the hemoglobin factor loadings matrix is not sparse enough. Algorithms Z and U depend less on sparsity and match the performance of algorithms F and W on this dataset. However, once we perform varimax rotation the performance of all the algorithms improves.

**Figure 8:**
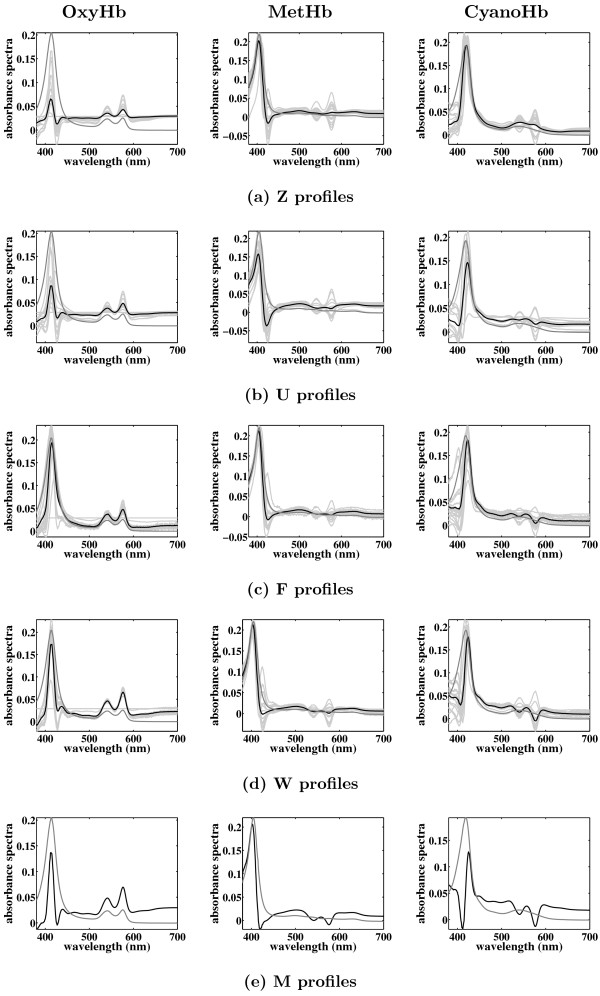
**Reconstruction of the factors matrix for the Hemoglobin data**. Shown are (a) the reconstructed profiles given by algorithm Z, (b) the reconstructed profiles given by algorithm U, (c) the reconstructed profiles given by algorithm F, (d) the reconstructed profiles given by algorithm W, and (e) the reconstructed profiles given by algorithm M. The positions of the zero entries in the loadings matrix are not given a priori. The light gray curves are the profiles given by the 20 different Gibbs sampling runs, and the black curves are the average profiles. We also plot with gray the true profiles for an easier comparison. These profiles are obtained after performing varimax rotation on the factor loadings matrix.

The classical FA algorithm (M) performs best according to the MSE of Λ. However, comparing the MSE of the factors (Figure 7(b)) its performance is worse. This is also apparent by looking at the factor profiles (Figure 8). Classical FA optimises the joint likelihood of the loadings matrix and noise covariance matrix (under a suitable constraint that guarantees identifiability), which amounts to integrating out the factors. All other algorithms (with the exception of Z) represent the factors explicitly. This explains why classical FA is doing better in reconstructing the loadings but worse in reconstructing factors compared to the other algorithms.

Algorithm F and W perform quite well on both the reconstruction of the Λ and the factor profiles. Their performance is also improved by using any of the four rotation methods. Varimax rotation also improves the performance of algorithms Z and U. Again procrustes rotation shows that we can rotate the estimated Λ to match the true Λ very closely. However, as shown again by the MSE on the factors, the best rotation for Λ is not necessarily the best rotation for the factors.

Figure 7(a) shows the result for algorithm F when entries of the loadings matrix are restricted to stay close to 0 by a strong prior (shape parameter 10 for *δ*_*pk*_, scale parameter 0.01, see methods section). We also investigated the performance of algorithm F under a vaguer prior on matrix entries (shape parameter 1 for *δ*_*pk*_, scale parameter 0.01). As shown in Figure 7(c) and 7(d), this setting (Fu) performs better, but once the loadings matrix is rotated, the improvement is not as significant. Similarly, we set the prior probability *π*_*pk *_(see methods section) that an entry of the loadings matrix is nonzero to 1 in algorithm W (Wu) and to 0.2 (W). As expected, since the connectivity is not sparse for the hemoglobin data, the difference is small (Figure 7(c)). With rotation (except the procrustes rotation) the sparse prior seems to do considerably better though. Finally, the algorithm Ws (with prior probability 0.2) that we have suggested in order to avoid the combinatorial problem of algorithm W gives good results and comparable to the ones by W.

Figure 8 shows the reconstructed factor profiles after varimax rotation on the Λ without using prior information on nonzero entries. It also demonstrates that it is now harder to reconstruct the factor profiles, as seen in the greater variability of profiles from different MCMC runs when compared to Figure 6. All the profiles shown have very similar likelihoods, indicating that the overall distribution is multimodal. Algorithms F and W perform quite well. Algorithms Z and U reconstruct the second and third factors quite well but not as well the first one. As mentioned above the reconstruction of the factor profiles by algorithm M are quite poor, while algorithm F seems to find the best factor profiles.

#### Escherichia coli dataset

We evaluated the FA algorithms as well as the algorithm by Boulesteix and Strimmer [8] (S, as implemented in the R package *plsgenomics*) on an *E. coli *dataset from Kao et al. [6]. These data consist of 25 time points for 100 genes. The first time point was ignored since all the values are zero. A matrix that indicates possible interactions between 16 TFs and the 100 genes has been suggested by Kao et al. [6] based on RegulonDB [18] and the current literature. We will refer to this matrix as the Kao connectivity matrix. This matrix also indicates whether a TF inhibits or activates a given gene. Each FA algorithm is run 10 times. The following results refer to an average value over these runs. The classical FA is not used in this analysis since the number of cases (24) is smaller than the number of observed variables (100).

Since the GNCA algorithm requires prior knowledge of zeros in the factor loadings matrix, for comparison we also run the FA algorithms of Fokoue [16] and West [3] providing prior information on zeros in the factor loadings matrix. Here, algorithms F and Ws treat the connectivity matrix simply as indicating whether there is a relationship or not between a gene and a TF and ignore the information on activation or inhibition. However, one could also include a more detailed prior information. We consider two different prior matrices for the GNCA algorithm: one where a simplified connectivity matrix that only indicates whether an interaction exists or not, and one with extra information on inhibition and activation. For each of the two different prior matrices, we run the GNCA algorithm 10 times, and we only consider the run with the least sum squared error.

**Figure 9:**
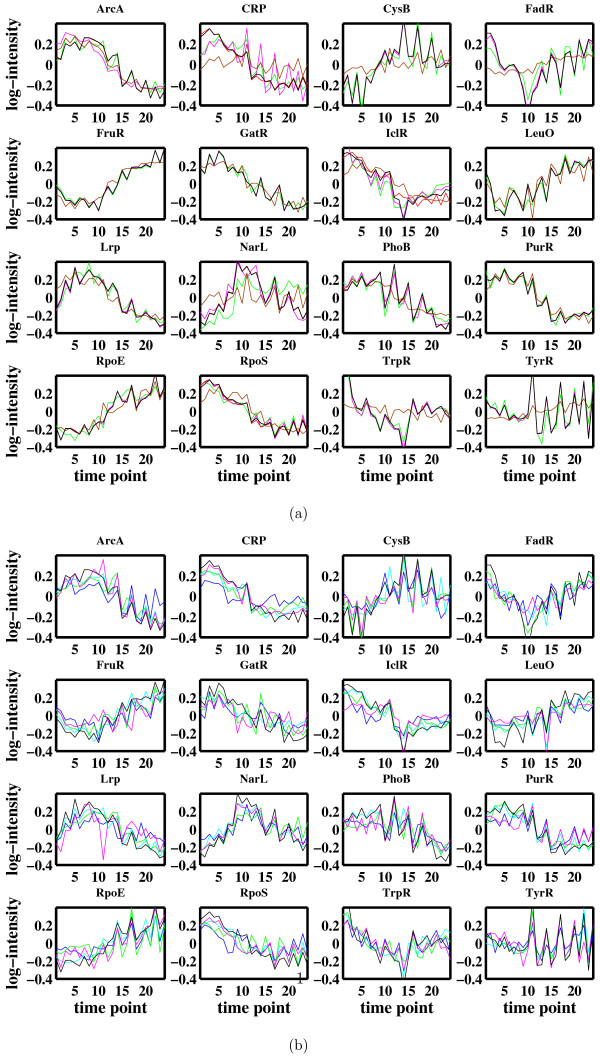
**Reconstruction of the factor profiles for the E. coli data**. a) prior connectivity structure is given and (b) no prior connectivity structure is given. Red lines correspond to algorithm GNCA, black lines correspond to GNCA where inhibition and activation information is also given, blue lines are for algorithm Z, cyan lines are for algorithm U, green lines correspond to algorithm F, purple lines are for algorithm Ws, and brown lines are for algorithm S.

Figure 9(a) shows that all the algorithms produce very similar TF profiles, that is, given the connectivity matrix, FA algorithms reconstruct TF profiles as well as GNCA. The second column in Table 1 shows the MSE deviation of profiles of algorithms F and Ws from profiles of GNCA which uses information on activation and inhibition. The MSE for GNCA is a consequence of using only connectivity information and no details on activation or inhibition. This small value of MSE suggests that convergence to a similar solution for the *E. coli *dataset is given regardless whether extra information on activation and inhibition is provided or not. The comparatively high MSE of algorithm S is due mainly to a few factors which are reconstructed as fiat.

**Table 1:** MSEs of the reconstructed factor profiles for the E. coli data

**algorithms**	**MSE (with prior information)**	**MSE (without prior information)**
Z	-	0.017
U	-	0.008
F	0.005	0.010
Ws	0.003	0.014
S	0.020	-
GNCA	0.0004	-

MSEs of the reconstructed factor matrices from the factor matrix obtained from GNCA with activation and inhibition information. The second column contains the MSEs when the zero positions in the loadings matrix are fixed. The third column contains the MSEs when no information regarding those positions is given. – indicates that the algorithm was not tested.

Figure 9(b) shows the results when no prior information on connectivity is provided. For comparison, we match the resulting TF profiles with those of GNCA by minimum MSE and add the plots of the GNCA TF profiles from Figure 9(a). As is evident, the FA algorithms in the case of the *E. coli *dataset are still capable of reconstructing important aspects of the TF profiles even without any prior information on the connectivity. This is encouraging since prior information on TF binding is sometimes limited, difficult to obtain, or not always reliable. The profiles are slightly rougher than the ones inferred given the connectivity matrix and the FA algorithms show greater variability. However, it is still impressive how all FA algorithms are able to reconstruct the main trends of the TF profiles. The third column in Table 1 shows the MSE deviation of profiles of algorithms Z, U, F and Ws from profiles of GNCA with activation and inhibition information. The MSE is about twice as large if no prior information is available.

**Figure 10:**
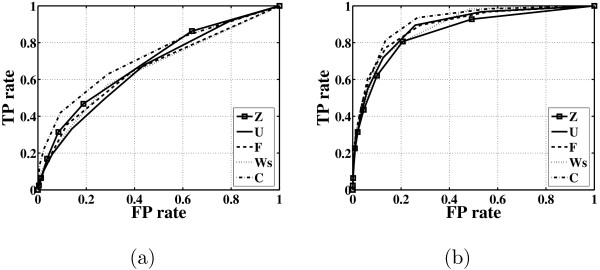
**Reconstruction of the factor loadings matrix for the E. coli data**. Shown for the *E. coli *dataset are (a) the ROC curve of each FA algorithm for the factor loadings matrix, and (b) the ROC curve of each FA algorithm for the factor loadings matrix after applying procrustes rotation method. The true positive (TP) rate is plotted against the false positive (FP) rate for a given cutoff value.

Finally, we analyse the inferred factor loadings matrix in greater detail. Such an evaluation is complicated by the fact that the true connectivity matrix is not fully known. For evaluating the learned loadings matrix, we treat the Kao connectivity matrix (Figure 1(a)) as showing true interactions and true missing interactions. However, we should keep in mind that the latter is based on partial biological information and not necessarily complete. Figure 10(a) shows a ROC curve for each algorithm. The true positive (TP) rate is the proportion of entries above a specified cutoff among entries which are nonzero according to the Kao connectivity matrix. The false positive (FP) rate is the proportion of entries above a specified cutoff among entries which are zero according to the Kao connectivity matrix. On average all algorithms give very similar performance. The lack of differences between the algorithms that implicitly consider sparsity, F and Ws, compared to the algorithms that do not, Z and U, could be due to the lack of detailed information in the Kao connectivity matrix. That is, this matrix has only 0,1 entries and actually some of the 1 entries could be very close to zero or exactly zero and in contrast some zero values could be nonzero. Figure 10(a) also shows a ROC curve that is based on merging the information gain by each algorithm. That is, we derive a combined factor loadings matrix by averaging the loading matrices derived by each algorithm. This combined loadings matrix gives a ROC curve that is better than any other ROC curve alone.

We also plot, in Figure 10(b), the ROC curve of each algorithm after applying procrustes rotation to the factor loadings matrix. Here, we use the Kao connectivity matrix as the target matrix for the procrustes rotation. The ROC curves have greatly improved indicating that an appropriate rotation of the learned loadings matrix for each algorithm can lead to a connectivity matrix that is very close to the Kao connectivity matrix. Again the combined loadings matrix gives a ROC curve that outperforms each of the ROC curves given by the FA algorithms.

## Conclusion

We discussed and compared the performance of five factor analysis algorithms presented previously in the literature. Only one of these algorithms has been previously applied to biological data. We investigated the applicability of the algorithms on microarray data from *E. coli*, on data from hemoglobin spectroscopic measurements and on simulated data. In a gene regulatory context, we aim to identify regulatory relationships between genes and TFs and to reconstruct transcription factor activity profiles. That is, the expression levels of regulated genes are the observed variables and the TFs are the unobserved variables. Even after imposing a correlation structure on the factors, this is still an underdetermined problem. If, however, we assume that the connectivity matrix is sparse, that is, that most genes are regulated by a small number of TFs and most TFs regulate only a small number of genes, estimation of TF profiles and loadings becomes possible.

The sparsity requirement is implicit in the algorithms by Fokoue [16] and West [3], and thus these algorithms are shown to perform very well on sparse simulated networks where the underlying relationships are linear. However, we show that the performance of the algorithms by Ghahramani and Hinton [13], and Utsugi and Kumagai [14] is also very satisfactory after an orthogonal rotation of the loadings matrix. On the *E. coli *data, we see that all the FA algorithms reconstruct the factor loadings matrix and the factors profiles equally well. Moreover, we show, using the *E. coli *data, that such algorithms can reconstruct the underlying TF profiles to an acceptable degree even without any prior knowledge of the connectivity structure. In contrast, algorithms such as the GNCA algorithm of Tran et al. [7], depend heavily on prior connectivity information. Finally, we show that integrating results from several FA algorithms results in a connectivity matrix which has a better true positive rate given a specified false positive rate than each algorithm separately. Our analysis demonstrates the usefulness of FA algorithms for biological problems where prior information regarding the system under study is not fully available.

The FA algorithms discussed here ignore any time series information. We are currently working on an extension of the above methods to integrate time correlation. We expect that such correlation will smooth TF activity profiles further.

## Methods

For completeness and to show commonalities and differences between the approaches to FA analysis discussed in this paper, we describe them in some detail in this section. We conclude this section with a short description of matrix rotation methods.

### Factors *F*

The factors are assumed to be normally distributed with mean zero and covariance matrix Σ*_f_*. That is,

*f*^*n *^~ N
 MathType@MTEF@5@5@+=feaafiart1ev1aaatCvAUfKttLearuWrP9MDH5MBPbIqV92AaeXatLxBI9gBaebbnrfifHhDYfgasaacH8akY=wiFfYdH8Gipec8Eeeu0xXdbba9frFj0=OqFfea0dXdd9vqai=hGuQ8kuc9pgc9s8qqaq=dirpe0xb9q8qiLsFr0=vr0=vr0dc8meaabaqaciaacaGaaeqabaqabeGadaaakeaat0uy0HwzTfgDPnwy1egaryqtHrhAL1wy0L2yHvdaiqaacqWFneVtaaa@383A@(0, Σ_*f*_)

To resolve identifiability problems (we will return to this issue later), we set Σ_*f *_equal to the identity matrix *I*_*K *_as suggested by Ghahramani and Hinton [13], Utsugi and Kumagai [14], and Fokoue [16]. Sabatti and James [4] choose Σ*f *= σf2
 MathType@MTEF@5@5@+=feaafiart1ev1aaatCvAUfKttLearuWrP9MDH5MBPbIqV92AaeXatLxBI9gBaebbnrfifHhDYfgasaacH8akY=wiFfYdH8Gipec8Eeeu0xXdbba9frFj0=OqFfea0dXdd9vqai=hGuQ8kuc9pgc9s8qqaq=dirpe0xb9q8qiLsFr0=vr0=vr0dc8meaabaqaciaacaGaaeqabaqabeGadaaakeaaiiGacqWFdpWCdaqhaaWcbaGaemOzaygabaGaeGOmaidaaaaa@30EA@*I*_*K *_where σf2
 MathType@MTEF@5@5@+=feaafiart1ev1aaatCvAUfKttLearuWrP9MDH5MBPbIqV92AaeXatLxBI9gBaebbnrfifHhDYfgasaacH8akY=wiFfYdH8Gipec8Eeeu0xXdbba9frFj0=OqFfea0dXdd9vqai=hGuQ8kuc9pgc9s8qqaq=dirpe0xb9q8qiLsFr0=vr0=vr0dc8meaabaqaciaacaGaaeqabaqabeGadaaakeaaiiGacqWFdpWCdaqhaaWcbaGaemOzaygabaGaeGOmaidaaaaa@30EA@ is a constant value. Finally, West [3] assigns a more general prior, Σ_*f *_= diag(σf12
 MathType@MTEF@5@5@+=feaafiart1ev1aaatCvAUfKttLearuWrP9MDH5MBPbIqV92AaeXatLxBI9gBaebbnrfifHhDYfgasaacH8akY=wiFfYdH8Gipec8Eeeu0xXdbba9frFj0=OqFfea0dXdd9vqai=hGuQ8kuc9pgc9s8qqaq=dirpe0xb9q8qiLsFr0=vr0=vr0dc8meaabaqaciaacaGaaeqabaqabeGadaaakeaaiiGacqWFdpWCdaqhaaWcbaGaemOzay2aaSbaaWqaaiabigdaXaqabaaaleaacqaIYaGmaaaaaa@3212@,..., σfK2
 MathType@MTEF@5@5@+=feaafiart1ev1aaatCvAUfKttLearuWrP9MDH5MBPbIqV92AaeXatLxBI9gBaebbnrfifHhDYfgasaacH8akY=wiFfYdH8Gipec8Eeeu0xXdbba9frFj0=OqFfea0dXdd9vqai=hGuQ8kuc9pgc9s8qqaq=dirpe0xb9q8qiLsFr0=vr0=vr0dc8meaabaqaciaacaGaaeqabaqabeGadaaakeaaiiGacqWFdpWCdaqhaaWcbaGaemOzay2aaSbaaWqaaiabdUealbqabaaaleaacqaIYaGmaaaaaa@3241@).

The posterior probability of the factors is now derived as

*p*(*f*^*n *^| *x*^*n*^, Λ, *μ*, Ψ) ∝ *p*(*f*^*n*^)*p*(*x*^*n *^| *f*^*n*^, Λ, *μ*, Ψ) = N
 MathType@MTEF@5@5@+=feaafiart1ev1aaatCvAUfKttLearuWrP9MDH5MBPbIqV92AaeXatLxBI9gBaebbnrfifHhDYfgasaacH8akY=wiFfYdH8Gipec8Eeeu0xXdbba9frFj0=OqFfea0dXdd9vqai=hGuQ8kuc9pgc9s8qqaq=dirpe0xb9q8qiLsFr0=vr0=vr0dc8meaabaqaciaacaGaaeqabaqabeGadaaakeaat0uy0HwzTfgDPnwy1egaryqtHrhAL1wy0L2yHvdaiqaacqWFneVtaaa@383A@(*f*^*n *^| mf*
 MathType@MTEF@5@5@+=feaafiart1ev1aaatCvAUfKttLearuWrP9MDH5MBPbIqV92AaeXatLxBI9gBaebbnrfifHhDYfgasaacH8akY=wiFfYdH8Gipec8Eeeu0xXdbba9frFj0=OqFfea0dXdd9vqai=hGuQ8kuc9pgc9s8qqaq=dirpe0xb9q8qiLsFr0=vr0=vr0dc8meaabaqaciaacaGaaeqabaqabeGadaaakeaacqWGTbqBdaqhaaWcbaGaemOzaygabaGaeiOkaOcaaaaa@306D@, Σf*
 MathType@MTEF@5@5@+=feaafiart1ev1aaatCvAUfKttLearuWrP9MDH5MBPbIqV92AaeXatLxBI9gBaebbnrfifHhDYfgasaacH8akY=wiFfYdH8Gipec8Eeeu0xXdbba9frFj0=OqFfea0dXdd9vqai=hGuQ8kuc9pgc9s8qqaq=dirpe0xb9q8qiLsFr0=vr0=vr0dc8meaabaqaciaacaGaaeqabaqabeGadaaakeaacqqHJoWudaqhaaWcbaGaemOzaygabaGaeiOkaOcaaaaa@308E@)

where the posterior mean and variance are given by

Σf*
 MathType@MTEF@5@5@+=feaafiart1ev1aaatCvAUfKttLearuWrP9MDH5MBPbIqV92AaeXatLxBI9gBaebbnrfifHhDYfgasaacH8akY=wiFfYdH8Gipec8Eeeu0xXdbba9frFj0=OqFfea0dXdd9vqai=hGuQ8kuc9pgc9s8qqaq=dirpe0xb9q8qiLsFr0=vr0=vr0dc8meaabaqaciaacaGaaeqabaqabeGadaaakeaacqqHJoWudaqhaaWcbaGaemOzaygabaGaeiOkaOcaaaaa@308E@ = (Σ_*f *_+ Λ'Ψ^-1^Λ)^-1^

mf*
 MathType@MTEF@5@5@+=feaafiart1ev1aaatCvAUfKttLearuWrP9MDH5MBPbIqV92AaeXatLxBI9gBaebbnrfifHhDYfgasaacH8akY=wiFfYdH8Gipec8Eeeu0xXdbba9frFj0=OqFfea0dXdd9vqai=hGuQ8kuc9pgc9s8qqaq=dirpe0xb9q8qiLsFr0=vr0=vr0dc8meaabaqaciaacaGaaeqabaqabeGadaaakeaacqWGTbqBdaqhaaWcbaGaemOzaygabaGaeiOkaOcaaaaa@306D@ = Σf*
 MathType@MTEF@5@5@+=feaafiart1ev1aaatCvAUfKttLearuWrP9MDH5MBPbIqV92AaeXatLxBI9gBaebbnrfifHhDYfgasaacH8akY=wiFfYdH8Gipec8Eeeu0xXdbba9frFj0=OqFfea0dXdd9vqai=hGuQ8kuc9pgc9s8qqaq=dirpe0xb9q8qiLsFr0=vr0=vr0dc8meaabaqaciaacaGaaeqabaqabeGadaaakeaacqqHJoWudaqhaaWcbaGaemOzaygabaGaeiOkaOcaaaaa@308E@Λ'Ψ^-1^(*x*^*n *^- *μ*)

We can now integrate *F *out of equation 4 to get the complete density of the data

p(X|Λ,μ,Ψ)=N(X|μ,ΛΣfΛ′+Ψ)=(2π)−N/2|ΛΣfΛ′+Ψ|−1/2×exp⁡(−12tr[(X−M)′(ΛΣfΛ′+Ψ)−1(X−M)])     (5)
 MathType@MTEF@5@5@+=feaafiart1ev1aaatCvAUfKttLearuWrP9MDH5MBPbIqV92AaeXatLxBI9gBaebbnrfifHhDYfgasaacH8akY=wiFfYdH8Gipec8Eeeu0xXdbba9frFj0=OqFfea0dXdd9vqai=hGuQ8kuc9pgc9s8qqaq=dirpe0xb9q8qiLsFr0=vr0=vr0dc8meaabaqaciaacaGaaeqabaqabeGadaaakeaafaqaaeGadaaabaGaemiCaaNaeiikaGIaemiwaGLaeiiFaWNaeu4MdWKaeiilaWccciGae8hVd0MaeiilaWIaeuiQdKLaeiykaKcabaGaeyypa0dabaWenfgDOvwBHrxAJfwnHbqeg0uy0HwzTfgDPnwy1aaceaGae4xdX7KaeiikaGIaemiwaGLaeiiFaWNae8hVd0MaeiilaWIaeu4MdWKaeu4Odm1aaSbaaSqaaiabdAgaMbqabaGccuqHBoatgaqbaiabgUcaRiabfI6azjabcMcaPaqaaaqaaiabg2da9aqaaiabcIcaOiabikdaYiab=b8aWjabcMcaPmaaCaaaleqabaGaeyOeI0IaemOta4Kaei4la8IaeGOmaidaaOGaeiiFaWNaeu4MdWKaeu4Odm1aaSbaaSqaaiabdAgaMbqabaGccuqHBoatgaqbaiabgUcaRiabfI6azjabcYha8naaCaaaleqabaGaeyOeI0IaeGymaeJaei4la8IaeGOmaidaaOGaey41aqRagiyzauMaeiiEaGNaeiiCaa3aaeWaaeaacqGHsisldaWcaaqaaiabigdaXaqaaiabikdaYaaacqqG0baDcqqGYbGCcqGGBbWwcqGGOaakcqWGybawcqGHsislcqWGnbqtcuGGPaqkgaqbaiabcIcaOiabfU5amjabfo6atnaaBaaaleaacqWGMbGzaeqaaOGafu4MdWKbauaacqGHRaWkcqqHOoqwcqGGPaqkdaahaaWcbeqaaiabgkHiTiabigdaXaaakiabcIcaOiabdIfayjabgkHiTiabd2eanjabcMcaPiabc2faDbGaayjkaiaawMcaaaaacaWLjaGaaCzcamaabmaabaGaeGynaudacaGLOaGaayzkaaaaaa@980D@

The EM algorithm of Ghahramani and Hinton [13] consists of two steps: a) the E-step which calculates the expected values and the second moments of the factors for each case *n *given the current Λ and Ψ as given below

E(fn|xn,Λ,Ψ)E(fn(fn)′|xn,Λ,Ψ)C  =Cxn=I−CΛ+Cxn(xn)′C′=Λ′(Ψ+ΛΛ′)−1
 MathType@MTEF@5@5@+=feaafiart1ev1aaatCvAUfKttLearuWrP9MDH5MBPbIqV92AaeXatLxBI9gBaebbnrfifHhDYfgasaacH8akY=wiFfYdH8Gipec8Eeeu0xXdbba9frFj0=OqFfea0dXdd9vqai=hGuQ8kuc9pgc9s8qqaq=dirpe0xb9q8qiLsFr0=vr0=vr0dc8meaabaqaciaacaGaaeqabaqabeGadaaakeaafaqaceWabaaabaGaemyrauKaeiikaGIaemOzay2aaWbaaSqabeaacqWGUbGBaaGccqGG8baFcqWG4baEdaahaaWcbeqaaiabd6gaUbaakiabcYcaSiabfU5amjabcYcaSiabfI6azjabcMcaPaqaaiabdweafjabcIcaOiabdAgaMnaaCaaaleqabaGaemOBa4gaaOGaeiikaGIaemOzay2aaWbaaSqabeaacqWGUbGBaaGccuGGPaqkgaqbaiabcYha8jabdIha4naaCaaaleqabaGaemOBa4gaaOGaeiilaWIaeu4MdWKaeiilaWIaeuiQdKLaeiykaKcabaGaem4qameaaiabbccaGiabbccaGuaabaqadiaaaeaacqGH9aqpaeaacqWGdbWqcqWG4baEdaahaaWcbeqaaiabd6gaUbaaaOqaaiabg2da9aqaaiabdMeajjabgkHiTiabdoeadjabfU5amjabgUcaRiabdoeadjabdIha4naaCaaaleqabaGaemOBa4gaaOGaeiikaGIaemiEaG3aaWbaaSqabeaacqWGUbGBaaGccuGGPaqkgaqbaiqbdoeadzaafaaabaGaeyypa0dabaGafu4MdWKbauaacqGGOaakcqqHOoqwcqGHRaWkcqqHBoatcuqHBoatgaqbaiabcMcaPmaaCaaaleqabaGaeyOeI0IaeGymaedaaaaaaaa@7392@

and b) the M-step which calculates the values of Λ and Ψ given the expected values of the factors that were computed in the E-step.

Λ=(∑n=1NxnE(fn|xn,Λ,Ψ)′)(∑i=1NE(fi(fi)′|xi,Λ,Ψ))−1Ψ=1Ndiag(∑n=1Nxn(xn)′−ΛE(fn|xn,Λ,Ψ)(xn)′)
 MathType@MTEF@5@5@+=feaafiart1ev1aaatCvAUfKttLearuWrP9MDH5MBPbIqV92AaeXatLxBI9gBaebbnrfifHhDYfgasaacH8akY=wiFfYdH8Gipec8Eeeu0xXdbba9frFj0=OqFfea0dXdd9vqai=hGuQ8kuc9pgc9s8qqaq=dirpe0xb9q8qiLsFr0=vr0=vr0dc8meaabaqaciaacaGaaeqabaqabeGadaaakeaafaqadeGabaaabaGaeu4MdWKaeyypa0ZaaeWaaeaadaaeWbqaaiabdIha4naaCaaaleqabaGaemOBa4gaaOGaemyrauKaeiikaGIaemOzay2aaWbaaSqabeaacqWGUbGBaaGccqGG8baFcqWG4baEdaahaaWcbeqaaiabd6gaUbaakiabcYcaSiabfU5amjabcYcaSiabfI6azjqbcMcaPyaafaaaleaacqWGUbGBcqGH9aqpcqaIXaqmaeaacqWGobGta0GaeyyeIuoaaOGaayjkaiaawMcaamaabmaabaWaaabCaeaacqWGfbqrcqGGOaakcqWGMbGzdaahaaWcbeqaaiabdMgaPbaakiabcIcaOiabdAgaMnaaCaaaleqabaGaemyAaKgaaOGafiykaKIbauaacqGG8baFcqWG4baEdaahaaWcbeqaaiabdMgaPbaakiabcYcaSiabfU5amjabcYcaSiabfI6azjabcMcaPaWcbaGaemyAaKMaeyypa0JaeGymaedabaGaemOta4eaniabggHiLdaakiaawIcacaGLPaaadaahaaWcbeqaaiabgkHiTiabigdaXaaaaOqaaiabfI6azjabg2da9maalaaabaGaeGymaedabaGaemOta4eaaiabbsgaKjabbMgaPjabbggaHjabbEgaNnaabmaabaWaaabCaeaacqWG4baEdaahaaWcbeqaaiabd6gaUbaakiabcIcaOiabdIha4naaCaaaleqabaGaemOBa4gaaOGafiykaKIbauaacqGHsislcqqHBoatcqWGfbqrcqGGOaakcqWGMbGzdaahaaWcbeqaaiabd6gaUbaakiabcYha8jabdIha4naaCaaaleqabaGaemOBa4gaaOGaeiilaWIaeu4MdWKaeiilaWIaeuiQdKLaeiykaKIaeiikaGIaemiEaG3aaWbaaSqabeaacqWGUbGBaaGccuGGPaqkgaqbaaWcbaGaemOBa4Maeyypa0JaeGymaedabaGaemOta4eaniabggHiLdaakiaawIcacaGLPaaaaaaaaa@983B@

### Mean vector *μ*

The prior probability assigned to the mean vector *μ *is the Gaussian distribution with a mean vector *m*_*μ *_and a covariance matrix Σ_*μ*_

*μ *~ N
 MathType@MTEF@5@5@+=feaafiart1ev1aaatCvAUfKttLearuWrP9MDH5MBPbIqV92AaeXatLxBI9gBaebbnrfifHhDYfgasaacH8akY=wiFfYdH8Gipec8Eeeu0xXdbba9frFj0=OqFfea0dXdd9vqai=hGuQ8kuc9pgc9s8qqaq=dirpe0xb9q8qiLsFr0=vr0=vr0dc8meaabaqaciaacaGaaeqabaqabeGadaaakeaat0uy0HwzTfgDPnwy1egaryqtHrhAL1wy0L2yHvdaiqaacqWFneVtaaa@383A@(*m*_*μ*_, Σ_*μ*_)

By using the above prior, we derive the following posterior distribution for *μ*

μΣμ∗mμ∗  ~N(mμ,Σμ∗)=(NΨ−1+Σμ−1)−1=Σμ∗(NΨ−1x¯+Σμ−1mμ)
 MathType@MTEF@5@5@+=feaafiart1ev1aaatCvAUfKttLearuWrP9MDH5MBPbIqV92AaeXatLxBI9gBaebbnrfifHhDYfgasaacH8akY=wiFfYdH8Gipec8Eeeu0xXdbba9frFj0=OqFfea0dXdd9vqai=hGuQ8kuc9pgc9s8qqaq=dirpe0xb9q8qiLsFr0=vr0=vr0dc8meaabaqaciaacaGaaeqabaqabeGadaaakeaafaqaceWabaaabaacciGae8hVd0gabaGaeu4Odm1aa0baaSqaaiab=X7aTbqaaiabgEHiQaaaaOqaaiabd2gaTnaaDaaaleaacqWF8oqBaeaacqGHxiIkaaaaaOGaeeiiaaIaeeiiaasbaeaabmGaaaqaaiabb6ha+bqaamrtHrhAL1wy0L2yHvtyaeHbnfgDOvwBHrxAJfwnaGabaiab+1q8ojabcIcaOiabd2gaTnaaBaaaleaacqWF8oqBaeqaaOGaeiilaWIaeu4Odm1aa0baaSqaaiab=X7aTbqaaiabgEHiQaaakiabcMcaPaqaaiabg2da9aqaaiabcIcaOiabd6eaojabfI6aznaaCaaaleqabaGaeyOeI0IaeGymaedaaOGaey4kaSIaeu4Odm1aa0baaSqaaiab=X7aTbqaaiabgkHiTiabigdaXaaakiabcMcaPmaaCaaaleqabaGaeyOeI0IaeGymaedaaaGcbaGaeyypa0dabaGaeu4Odm1aa0baaSqaaiab=X7aTbqaaiabgEHiQaaakiabcIcaOiabd6eaojabfI6aznaaCaaaleqabaGaeyOeI0IaeGymaedaaOGafmiEaGNbaebacqGHRaWkcqqHJoWudaqhaaWcbaGae8hVd0gabaGaeyOeI0IaeGymaedaaOGaemyBa02aaSbaaSqaaiab=X7aTbqabaGccqGGPaqkaaaaaa@7695@

where

x¯=1N∑n=1N(xn−Λfn)
 MathType@MTEF@5@5@+=feaafiart1ev1aaatCvAUfKttLearuWrP9MDH5MBPbIqV92AaeXatLxBI9gBaebbnrfifHhDYfgasaacH8akY=wiFfYdH8Gipec8Eeeu0xXdbba9frFj0=OqFfea0dXdd9vqai=hGuQ8kuc9pgc9s8qqaq=dirpe0xb9q8qiLsFr0=vr0=vr0dc8meaabaqaciaacaGaaeqabaqabeGadaaakeaacuWG4baEgaqeaiabg2da9maalaaabaGaeGymaedabaGaemOta4eaamaaqahabaGaeiikaGIaemiEaG3aaWbaaSqabeaacqWGUbGBaaGccqGHsislcqqHBoatcqWGMbGzdaahaaWcbeqaaiabd6gaUbaakiabcMcaPaWcbaGaemOBa4Maeyypa0JaeGymaedabaGaemOta4eaniabggHiLdaaaa@4244@

West [3], Sabatti and James [4], and Fokoue [16] suggest to centralise the data prior to the use of the FA model, and they also assume that *μ *= 0. We also suggest to standardise (centralise and scale by standard deviation) the data prior to the analysis.

Utsugi and Kumagai [14] use a different prior covariance matrix that ties the mean to the error term. That is,

μ~N(mμ,α1−1Ψ)
 MathType@MTEF@5@5@+=feaafiart1ev1aaatCvAUfKttLearuWrP9MDH5MBPbIqV92AaeXatLxBI9gBaebbnrfifHhDYfgasaacH8akY=wiFfYdH8Gipec8Eeeu0xXdbba9frFj0=OqFfea0dXdd9vqai=hGuQ8kuc9pgc9s8qqaq=dirpe0xb9q8qiLsFr0=vr0=vr0dc8meaabaqaciaacaGaaeqabaqabeGadaaakeaaiiGacqWF8oqBcqGG+bGFt0uy0HwzTfgDPnwy1egaryqtHrhAL1wy0L2yHvdaiqaacqGFneVtcqGGOaakcqWGTbqBdaWgaaWcbaGae8hVd0gabeaakiabcYcaSiab=f7aHnaaDaaaleaacqaIXaqmaeaacqGHsislcqaIXaqmaaGccqqHOoqwcqGGPaqkaaa@4783@

where *m*_*μ *_is set to zero since they also centralize the data prior to the use of the FA model. The posterior distribution of *μ*, given the above prior is derived in the next section together with Λ.

### Factor loadings matrix Λ

The main differences between the existing FA models lies in the assignment of the prior distribution of the factor loadings matrix Λ or in the prior distribution of its parameters. Let us discuss each of these priors separately.

#### Normal prior on Λ and Gamma prior on Λ's covariance parameter

Fokoue [16] uses the prior suggested by Tipping [20] in the context of *Relevance Vector Machines *to impose sparsity in the Λ matrix. That is, independent Gaussian priors are assigned to each element *λ*_*pk *_of Λ.

*λ*_*pk *_| *δ*_*pk *_~ N
 MathType@MTEF@5@5@+=feaafiart1ev1aaatCvAUfKttLearuWrP9MDH5MBPbIqV92AaeXatLxBI9gBaebbnrfifHhDYfgasaacH8akY=wiFfYdH8Gipec8Eeeu0xXdbba9frFj0=OqFfea0dXdd9vqai=hGuQ8kuc9pgc9s8qqaq=dirpe0xb9q8qiLsFr0=vr0=vr0dc8meaabaqaciaacaGaaeqabaqabeGadaaakeaat0uy0HwzTfgDPnwy1egaryqtHrhAL1wy0L2yHvdaiqaacqWFneVtaaa@383A@(0, δpk−1
 MathType@MTEF@5@5@+=feaafiart1ev1aaatCvAUfKttLearuWrP9MDH5MBPbIqV92AaeXatLxBI9gBaebbnrfifHhDYfgasaacH8akY=wiFfYdH8Gipec8Eeeu0xXdbba9frFj0=OqFfea0dXdd9vqai=hGuQ8kuc9pgc9s8qqaq=dirpe0xb9q8qiLsFr0=vr0=vr0dc8meaabaqaciaacaGaaeqabaqabeGadaaakeaaiiGacqWF0oazdaqhaaWcbaGaemiCaaNaem4AaSgabaGaeyOeI0IaeGymaedaaaaa@332A@)

To each *δ*_*pk*_, a Gamma prior is assigned as follows

*δ*_*pk *_| *α*_*δ*_, *β*_*δ *_~ G
 MathType@MTEF@5@5@+=feaafiart1ev1aaatCvAUfKttLearuWrP9MDH5MBPbIqV92AaeXatLxBI9gBaebbnrfifHhDYfgasaacH8akY=wiFfYdH8Gipec8Eeeu0xXdbba9frFj0=OqFfea0dXdd9vqai=hGuQ8kuc9pgc9s8qqaq=dirpe0xb9q8qiLsFr0=vr0=vr0dc8meaabaqaciaacaGaaeqabaqabeGadaaakeaat0uy0HwzTfgDPnwy1egaryqtHrhAL1wy0L2yHvdaiqaacqWFge=raaa@382C@(*α*_*δ*_, *β*_*δ*_)

where a Gamma distribution with shape parameter *α *and a scale parameter *β *is defined as

p(x)=xα−1βαΓ(α)e−βx
 MathType@MTEF@5@5@+=feaafiart1ev1aaatCvAUfKttLearuWrP9MDH5MBPbIqV92AaeXatLxBI9gBaebbnrfifHhDYfgasaacH8akY=wiFfYdH8Gipec8Eeeu0xXdbba9frFj0=OqFfea0dXdd9vqai=hGuQ8kuc9pgc9s8qqaq=dirpe0xb9q8qiLsFr0=vr0=vr0dc8meaabaqaciaacaGaaeqabaqabeGadaaakeaacqWGWbaCcqGGOaakcqWG4baEcqGGPaqkcqGH9aqpdaWcaaqaaiabdIha4naaCaaaleqabaacciGae8xSdeMaeyOeI0IaeGymaedaaOGae8NSdi2aaWbaaSqabeaacqWFXoqyaaaakeaacqqHtoWrcqGGOaakcqWFXoqycqGGPaqkaaGaemyzau2aaWbaaSqabeaacqGHsislcqWFYoGycqWG4baEaaaaaa@452C@

In the context of biological data, we suspect that each gene is regulated by only a small number of TFs. Thus, we aim to identify a sparse factor loadings matrix that faithfully describes the relationship between the transcription factors and the regulated genes. The suggested prior leads to a Student *t*-distribution for each row of Λ. In two dimensions, such distribution assigns most probability mass to the origin where both *λ*_*p*1 _and *λ*_*p*2 _are zero and along the spines where one of the coefficients *λ*_*pk *_is zero.

We suggest that this type of prior on Λ is also applicable to biological data. We have also further extended this prior to include an extra level of hyperparameters for increased flexibility and for an easier assignment of the hyperparameters. Thus, the parameter *β*_*δ *_has also a Gamma prior of the form

βδ|αβδ,ββδ~G(αβδ,ββδ)
 MathType@MTEF@5@5@+=feaafiart1ev1aaatCvAUfKttLearuWrP9MDH5MBPbIqV92AaeXatLxBI9gBaebbnrfifHhDYfgasaacH8akY=wiFfYdH8Gipec8Eeeu0xXdbba9frFj0=OqFfea0dXdd9vqai=hGuQ8kuc9pgc9s8qqaq=dirpe0xb9q8qiLsFr0=vr0=vr0dc8meaabaqaciaacaGaaeqabaqabeGadaaakeaaiiGacqWFYoGydaWgaaWcbaGae8hTdqgabeaakiabcYha8jab=f7aHnaaBaaaleaacqWFYoGydaWgaaadbaGae8hTdqgabeaaaSqabaGccqGGSaalcqWFYoGydaWgaaWcbaGae8NSdi2aaSbaaWqaaiab=r7aKbqabaaaleqaaOGaeiOFa43enfgDOvwBHrxAJfwnHbqeg0uy0HwzTfgDPnwy1aaceaGae4NbXFKaeiikaGIae8xSde2aaSbaaSqaaiab=j7aInaaBaaameaacqWF0oazaeqaaaWcbeaakiabcYcaSiab=j7aInaaBaaaleaacqWFYoGydaWgaaadbaGae8hTdqgabeaaaSqabaGccqGGPaqkaaa@5733@

The posterior probability of each row Λ_*p *_of Λ is given by

*p*(Λ_*p *_| *X*, *F*, *μ*, Ψ, Δ_*p*_) ∝ *p*(Λ_*p *_| Δ_*p*_)*p*(*X *| *F*, Λ, *μ*, Ψ) = N
 MathType@MTEF@5@5@+=feaafiart1ev1aaatCvAUfKttLearuWrP9MDH5MBPbIqV92AaeXatLxBI9gBaebbnrfifHhDYfgasaacH8akY=wiFfYdH8Gipec8Eeeu0xXdbba9frFj0=OqFfea0dXdd9vqai=hGuQ8kuc9pgc9s8qqaq=dirpe0xb9q8qiLsFr0=vr0=vr0dc8meaabaqaciaacaGaaeqabaqabeGadaaakeaat0uy0HwzTfgDPnwy1egaryqtHrhAL1wy0L2yHvdaiqaacqWFneVtaaa@383A@(Λ_*p *_| mΛp
 MathType@MTEF@5@5@+=feaafiart1ev1aaatCvAUfKttLearuWrP9MDH5MBPbIqV92AaeXatLxBI9gBaebbnrfifHhDYfgasaacH8akY=wiFfYdH8Gipec8Eeeu0xXdbba9frFj0=OqFfea0dXdd9vqai=hGuQ8kuc9pgc9s8qqaq=dirpe0xb9q8qiLsFr0=vr0=vr0dc8meaabaqaciaacaGaaeqabaqabeGadaaakeaacqWGTbqBdaWgaaWcbaGaeu4MdW0aaSbaaWqaaiabdchaWbqabaaaleqaaaaa@3151@, ΣΛp
 MathType@MTEF@5@5@+=feaafiart1ev1aaatCvAUfKttLearuWrP9MDH5MBPbIqV92AaeXatLxBI9gBaebbnrfifHhDYfgasaacH8akY=wiFfYdH8Gipec8Eeeu0xXdbba9frFj0=OqFfea0dXdd9vqai=hGuQ8kuc9pgc9s8qqaq=dirpe0xb9q8qiLsFr0=vr0=vr0dc8meaabaqaciaacaGaaeqabaqabeGadaaakeaacqqHJoWudaWgaaWcbaGaeu4MdW0aaSbaaWqaaiabdchaWbqabaaaleqaaaaa@3172@)   (7)

where

ΣΛp=(ψp−2FF′+Δp−1)−1mΛp=ΣΛpF(Xp−Mp)′ψp−2
 MathType@MTEF@5@5@+=feaafiart1ev1aaatCvAUfKttLearuWrP9MDH5MBPbIqV92AaeXatLxBI9gBaebbnrfifHhDYfgasaacH8akY=wiFfYdH8Gipec8Eeeu0xXdbba9frFj0=OqFfea0dXdd9vqai=hGuQ8kuc9pgc9s8qqaq=dirpe0xb9q8qiLsFr0=vr0=vr0dc8meaabaqaciaacaGaaeqabaqabeGadaaakeaafaqadeGabaaabaGaeu4Odm1aaSbaaSqaaiabfU5amnaaBaaameaacqWGWbaCaeqaaaWcbeaakiabg2da9iabcIcaOGGaciab=H8a5naaDaaaleaacqWGWbaCaeaacqGHsislcqaIYaGmaaGccqWGgbGrcuWGgbGrgaqbaiabgUcaRiabfs5aenaaDaaaleaacqWGWbaCaeaacqGHsislcqaIXaqmaaGccqGGPaqkdaahaaWcbeqaaiabgkHiTiabigdaXaaaaOqaaiabd2gaTnaaBaaaleaacqqHBoatdaWgaaadbaGaemiCaahabeaaaSqabaGccqGH9aqpcqqHJoWudaWgaaWcbaGaeu4MdW0aaSbaaWqaaiabdchaWbqabaaaleqaaOGaemOrayKaeiikaGIaemiwaG1aaSbaaSqaaiabdchaWbqabaGccqGHsislcqWGnbqtdaWgaaWcbaGaemiCaahabeaakiqbcMcaPyaafaGae8hYdK3aa0baaSqaaiabdchaWbqaaiabgkHiTiabikdaYaaaaaaaaa@5CC3@

Δ_*p *_= diag(δp1−1
 MathType@MTEF@5@5@+=feaafiart1ev1aaatCvAUfKttLearuWrP9MDH5MBPbIqV92AaeXatLxBI9gBaebbnrfifHhDYfgasaacH8akY=wiFfYdH8Gipec8Eeeu0xXdbba9frFj0=OqFfea0dXdd9vqai=hGuQ8kuc9pgc9s8qqaq=dirpe0xb9q8qiLsFr0=vr0=vr0dc8meaabaqaciaacaGaaeqabaqabeGadaaakeaaiiGacqWF0oazdaqhaaWcbaGaemiCaaNaeGymaedabaGaeyOeI0IaeGymaedaaaaa@32BB@,... ,δpK−1
 MathType@MTEF@5@5@+=feaafiart1ev1aaatCvAUfKttLearuWrP9MDH5MBPbIqV92AaeXatLxBI9gBaebbnrfifHhDYfgasaacH8akY=wiFfYdH8Gipec8Eeeu0xXdbba9frFj0=OqFfea0dXdd9vqai=hGuQ8kuc9pgc9s8qqaq=dirpe0xb9q8qiLsFr0=vr0=vr0dc8meaabaqaciaacaGaaeqabaqabeGadaaakeaaiiGacqWF0oazdaqhaaWcbaGaemiCaaNaem4saSeabaGaeyOeI0IaeGymaedaaaaa@32EA@), and *A*_*p *_is a row vector that corresponds to the *p*^*th *^row of *A*.

The posterior distribution of *δ*_*pk *_is also a Gamma distribution given by

p(δpk|λpk)∝(δpk|αδ,βδ)p(λpk|δpk)=G(δpk|αδ+12,βδ+λpk22)
 MathType@MTEF@5@5@+=feaafiart1ev1aaatCvAUfeBSjuyZL2yd9gzLbvyNv2CaerbwvMCKfMBHbqedmvETj2BSbWenfgDOvwBHrxAJfwnHbqeg0uy0HwzTfgDPnwy1aqee0evGueE0jxyaibaieIgFLIOYR2NHOxjYhrPYhrPYpI8F4rqqrFfpeea0xe9Lq=Jc9vqaqpepm0xbbG8FasPYRqj0=yi0lXdbba9pGe9qqFf0dXdHuk9fr=xfr=xfrpiWZqaaeaabiGaaiaacaqabeaadaqacqaaaOqaaiaadchacaGGOaacciGae8hTdq2aaSbaaSqaaiaadchacaWGRbaabeaakiaacYhacqWF7oaBdaWgaaWcbaGaamiCaiaadUgaaeqaaOGaaiykaiabg2Hi1kaacIcacqWF0oazdaWgaaWcbaGaamiCaiaadUgaaeqaaOGaaiiFaiab=f7aHnaaBaaaleaacqWF0oazaeqaaOGaaiilaiab=j7aInaaBaaaleaacqWF0oazaeqaaOGaaiykaiaadchacaGGOaGae83UdW2aaSbaaSqaaiaadchacaWGRbaabeaakiaacYhacqWF0oazdaWgaaWcbaGaamiCaiaadUgaaeqaaOGaaiykaiabg2da9GWaaiab+zq8hnaabmaabaGae8hTdq2aaSbaaSqaaiaadchacaWGRbaabeaakiaacYhacqWFXoqydaWgaaWcbaGae8hTdqgabeaakiabgUcaRmaalaaabaGaaGymaaqaaiaaikdaaaGaaiilaiab=j7aInaaBaaaleaacqWF0oazaeqaaOGaey4kaSYaaSaaaeaacqWF7oaBdaqhaaWcbaGaamiCaiaadUgaaeaacaaIYaaaaaGcbaGaaGOmaaaaaiaawIcacaGLPaaaaaa@7D4F@

Finally, the posterior distribution of the scale parameter *β*_*δ *_of Δ is given by

p(βδ|Δ)∝p(βδ|αβδ,ββδ)p(Δ)=G(βδ|αβδ+PKαδ,ββδ+∑p=1P∑k=1Kδpk−1)
 MathType@MTEF@5@5@+=feaafiart1ev1aaatCvAUfKttLearuWrP9MDH5MBPbIqV92AaeXatLxBI9gBaebbnrfifHhDYfgasaacH8akY=wiFfYdH8Gipec8Eeeu0xXdbba9frFj0=OqFfea0dXdd9vqai=hGuQ8kuc9pgc9s8qqaq=dirpe0xb9q8qiLsFr0=vr0=vr0dc8meaabaqaciaacaGaaeqabaqabeGadaaakeaacqWGWbaCcqGGOaakiiGacqWFYoGydaWgaaWcbaGae8hTdqgabeaakiabcYha8jabfs5aejabcMcaPiabg2Hi1kabdchaWjabcIcaOiab=j7aInaaBaaaleaacqWF0oazaeqaaOGaeiiFaWNae8xSde2aaSbaaSqaaiab=j7aInaaBaaameaacqWF0oazaeqaaaWcbeaakiabcYcaSiab=j7aInaaBaaaleaacqWFYoGydaWgaaadbaGae8hTdqgabeaaaSqabaGccqGGPaqkcqWGWbaCcqGGOaakcqqHuoarcqGGPaqkcqGH9aqpt0uy0HwzTfgDPnwy1egaryqtHrhAL1wy0L2yHvdaiqaacqGFge=rdaqadaqaaiab=j7aInaaBaaaleaacqWF0oazaeqaaOGaeiiFaWNae8xSde2aaSbaaSqaaiab=j7aInaaBaaameaacqWF0oazaeqaaaWcbeaakiabgUcaRiabdcfaqjabdUealjab=f7aHnaaBaaaleaacqWF0oazaeqaaOGaeiilaWIae8NSdi2aaSbaaSqaaiab=j7aInaaBaaameaacqWF0oazaeqaaaWcbeaakiabgUcaRmaaqahabaWaaabCaeaacqWF0oazdaqhaaWcbaGaemiCaaNaem4AaSgabaGaeyOeI0IaeGymaedaaaqaaiabdUgaRjabg2da9iabigdaXaqaaiabdUealbqdcqGHris5aaWcbaGaemiCaaNaeyypa0JaeGymaedabaGaemiuaafaniabggHiLdaakiaawIcacaGLPaaaaaa@897A@

#### Mixture prior on Λ

West [3] has suggested a mixture prior on the elements *λ*_*pk *_that also induces sparsity on the factor loadings matrix Λ. Thus each element *λ*_*pk *_has the following prior

p(λpk)=(1−πpk)δ0(λpk)+πpkN(λpk|0,σΛ2)
 MathType@MTEF@5@5@+=feaafiart1ev1aaatCvAUfKttLearuWrP9MDH5MBPbIqV92AaeXatLxBI9gBaebbnrfifHhDYfgasaacH8akY=wiFfYdH8Gipec8Eeeu0xXdbba9frFj0=OqFfea0dXdd9vqai=hGuQ8kuc9pgc9s8qqaq=dirpe0xb9q8qiLsFr0=vr0=vr0dc8meaabaqaciaacaGaaeqabaqabeGadaaakeaacqWGWbaCcqGGOaakiiGacqWF7oaBdaWgaaWcbaGaemiCaaNaem4AaSgabeaakiabcMcaPiabg2da9iabcIcaOiabigdaXiabgkHiTiab=b8aWnaaBaaaleaacqWGWbaCcqWGRbWAaeqaaOGaeiykaKIae8hTdq2aaSbaaSqaaiabicdaWaqabaGccqGGOaakcqWF7oaBdaWgaaWcbaGaemiCaaNaem4AaSgabeaakiabcMcaPiabgUcaRiab=b8aWnaaBaaaleaacqWGWbaCcqWGRbWAaeqaamrtHrhAL1wy0L2yHvtyaeHbnfgDOvwBHrxAJfwnaGabaOGae4xdX7KaeiikaGIae83UdW2aaSbaaSqaaiabdchaWjabdUgaRbqabaGccqGG8baFcqaIWaamcqGGSaalcqWFdpWCdaqhaaWcbaGaeu4MdWeabaGaeGOmaidaaOGaeiykaKcaaa@661C@

where *δ*_0 _is the unit point mass at zero, and *π*_*pk *_indicates the probability of *λ*_*pk *_to be different from zero. We set *π*_*pk *_to 0.2 in the case of unknown connectivity and to 0 and 1 in the case the connectivity is known. An auxiliary variable is usually used to enable the calculation of the posterior probabilities. Thus, let us introduce a matrix of indicator variables *Z *with each element *z*_*pk*_, corresponding to each element *λ*_*pk*_. The prior probability on *Z *is a product of independent Bernoulli distributions as follows

p(Z)=∏p=1P∏k=1Kπpkzpk(1−πpk)1−zpk
 MathType@MTEF@5@5@+=feaafiart1ev1aaatCvAUfKttLearuWrP9MDH5MBPbIqV92AaeXatLxBI9gBaebbnrfifHhDYfgasaacH8akY=wiFfYdH8Gipec8Eeeu0xXdbba9frFj0=OqFfea0dXdd9vqai=hGuQ8kuc9pgc9s8qqaq=dirpe0xb9q8qiLsFr0=vr0=vr0dc8meaabaqaciaacaGaaeqabaqabeGadaaakeaacqWGWbaCcqGGOaakcqWGAbGwcqGGPaqkcqGH9aqpdaqeWbqaamaarahabaacciGae8hWda3aa0baaSqaaiabdchaWjabdUgaRbqaaiabdQha6naaBaaameaacqWGWbaCcqWGRbWAaeqaaaaaaSqaaiabdUgaRjabg2da9iabigdaXaqaaiabdUealbqdcqGHpis1aaWcbaGaemiCaaNaeyypa0JaeGymaedabaGaemiuaafaniabg+GivdGccqGGOaakcqaIXaqmcqGHsislcqWFapaCdaWgaaWcbaGaemiCaaNaem4AaSgabeaakiabcMcaPmaaCaaaleqabaGaeGymaeJaeyOeI0IaemOEaO3aaSbaaWqaaiabdchaWjabdUgaRbqabaaaaaaa@575E@

The *z*_*pk *_variables are called *indicators*, since they indicate whether the value of *λ*_*pk *_is to be drawn from the normal distribution or set to zero. That is,

λpk|zpk=0~δ0λpk|zpk=1~N(0,σλ2)
 MathType@MTEF@5@5@+=feaafiart1ev1aaatCvAUfKttLearuWrP9MDH5MBPbIqV92AaeXatLxBI9gBaebbnrfifHhDYfgasaacH8akY=wiFfYdH8Gipec8Eeeu0xXdbba9frFj0=OqFfea0dXdd9vqai=hGuQ8kuc9pgc9s8qqaq=dirpe0xb9q8qiLsFr0=vr0=vr0dc8meaabaqaciaacaGaaeqabaqabeGadaaakeaafaqadeGabaaabaacciGae83UdW2aaSbaaSqaaiabdchaWjabdUgaRbqabaGccqGG8baFcqWG6bGEdaWgaaWcbaGaemiCaaNaem4AaSgabeaakiabg2da9iabicdaWiabc6ha+jab=r7aKnaaBaaaleaacqaIWaamaeqaaaGcbaGae83UdW2aaSbaaSqaaiabdchaWjabdUgaRbqabaGccqGG8baFcqWG6bGEdaWgaaWcbaGaemiCaaNaem4AaSgabeaakiabg2da9iabigdaXiabc6ha+nrtHrhAL1wy0L2yHvtyaeHbnfgDOvwBHrxAJfwnaGabaiab+1q8ojabcIcaOiabicdaWiabcYcaSiab=n8aZnaaDaaaleaacqWF7oaBaeaacqWFYaGmaaGccqGGPaqkaaaaaa@5F67@

The posterior probability of the vector variable *Z*_*p *_= (*z*_*p*1_,..., *z*_*pK*_) does not have a known form (see equation 8). Thus we have to calculate equation 8 for all possible configurations of *Z*_*p *_and then use the multinomial probability distribution to sample a new configuration for *Z*_*p*_. This is a combinatorial problem, and thus as the number of hidden variables increases, the computational cost increases exponentially.

p(Zp)=∏k=1Kπpkzpk(1−πpk)1−zpk×σλ−|Zp|det⁡(ψp−2F[Zp]F[Zp]′+σλ−2IK′)−1/2×exp⁡(12ψp4XpF[Zp]′(ψp−2F[Zp]F[Zp]′+σλ−2IK′)−1F[Zp]X′p)     (8)
 MathType@MTEF@5@5@+=feaafiart1ev1aaatCvAUfKttLearuWrP9MDH5MBPbIqV92AaeXatLxBI9gBaebbnrfifHhDYfgasaacH8akY=wiFfYdH8Gipec8Eeeu0xXdbba9frFj0=OqFfea0dXdd9vqai=hGuQ8kuc9pgc9s8qqaq=dirpe0xb9q8qiLsFr0=vr0=vr0dc8meaabaqaciaacaGaaeqabaqabeGadaaakeaafaqadeGadaaabaGaemiCaaNaeiikaGIaemOwaO1aaSbaaSqaaiabdchaWbqabaGccqGGPaqkaeaacqGH9aqpaeaadaqeWbqaaGGaciab=b8aWnaaDaaaleaacqWGWbaCcqWGRbWAaeaacqWG6bGEdaWgaaadbaGaemiCaaNaem4AaSgabeaaaaaaleaacqWGRbWAcqGH9aqpcqaIXaqmaeaacqWGlbWsa0Gaey4dIunakiabcIcaOiabigdaXiabgkHiTiab=b8aWnaaBaaaleaacqWGWbaCcqWGRbWAaeqaaOGaeiykaKYaaWbaaSqabeaacqaIXaqmcqGHsislcqWG6bGEdaWgaaadbaGaemiCaaNaem4AaSgabeaaaaGccqGHxdaTcqWFdpWCdaqhaaWcbaGae83UdWgabaGaeyOeI0IaeiiFaWNaemOwaO1aaSbaaWqaaiabdchaWbqabaWccqGG8baFaaGccyGGKbazcqGGLbqzcqGG0baDcqGGOaakcqWFipqEdaqhaaWcbaGaemiCaahabaGaeyOeI0IaeGOmaidaaOGaemOrayKaei4waSLaemOwaO1aaSbaaSqaaiabdchaWbqabaGccqGGDbqxcqWGgbGrcqGGBbWwcqWGAbGwdaWgaaWcbaGaemiCaahabeaakiqbc2faDzaafaGaey4kaSIae83Wdm3aa0baaSqaaiab=T7aSbqaaiabgkHiTiabikdaYaaakiabdMeajnaaBaaaleaacuWGlbWsgaqbaaqabaGccqGGPaqkdaahaaWcbeqaaiabgkHiTiabigdaXiabc+caViabikdaYaaaaOqaaaqaaiabgEna0cqaaiGbcwgaLjabcIha4jabcchaWnaabmaabaWaaSaaaeaacqaIXaqmaeaacqaIYaGmcqWFipqEdaqhaaWcbaGaemiCaahabaGaeGinaqdaaaaakiabdIfaynaaBaaaleaacqWGWbaCaeqaaOGaemOrayKaei4waSLaemOwaO1aaSbaaSqaaiabdchaWbqabaGccuGGDbqxgaqbaiabcIcaOiab=H8a5naaDaaaleaacqWGWbaCaeaacqGHsislcqaIYaGmaaGccqWGgbGrcqGGBbWwcqWGAbGwdaWgaaWcbaGaemiCaahabeaakiabc2faDjabdAeagjabcUfaBjabdQfaAnaaBaaaleaacqWGWbaCaeqaaOGafiyxa0LbauaacqGHRaWkcqWFdpWCdaqhaaWcbaGae83UdWgabaGaeyOeI0IaeGOmaidaaOGaemysaK0aaSbaaSqaaiqbdUealzaafaaabeaakiabcMcaPmaaCaaaleqabaGaeyOeI0IaeGymaedaaOGaemOrayKaei4waSLaemOwaO1aaSbaaSqaaiabdchaWbqabaGccqGGDbqxcuWGybawgaqbamaaBaaaleaacqWGWbaCaeqaaaGccaGLOaGaayzkaaaaaiaaxMaacaWLjaWaaeWaaeaacqaI4aaoaiaawIcacaGLPaaaaaa@C729@

where *F*[*Z*_*p*_] denotes the submatrix of *F *obtained by removing those rows of *F *corresponding to *z*_*pk *_= 0, *K*' is the number of factors for which *z*_*pk *_= 1, and *I*_*K*' _is the identity matrix of *K*' dimensions. We also tested a version Ws of this algorithm in which equation 8 (with *K *= 1) is applied to each entry of the matrix individually, that is, without the need of a combinatorial evaluation of all possible 0,1 vectors *Z*_*p*_.

The posterior distribution of each row Λ_*p *_of Λ is the same as in equation 7 but *F *is now replaced by *F*[*Z*_*p*_] and Δp−1
 MathType@MTEF@5@5@+=feaafiart1ev1aaatCvAUfKttLearuWrP9MDH5MBPbIqV92AaeXatLxBI9gBaebbnrfifHhDYfgasaacH8akY=wiFfYdH8Gipec8Eeeu0xXdbba9frFj0=OqFfea0dXdd9vqai=hGuQ8kuc9pgc9s8qqaq=dirpe0xb9q8qiLsFr0=vr0=vr0dc8meaabaqaciaacaGaaeqabaqabeGadaaakeaacqqHuoardaqhaaWcbaGaemiCaahabaGaeyOeI0IaeGymaedaaaaa@3185@ by σλ−2
 MathType@MTEF@5@5@+=feaafiart1ev1aaatCvAUfKttLearuWrP9MDH5MBPbIqV92AaeXatLxBI9gBaebbnrfifHhDYfgasaacH8akY=wiFfYdH8Gipec8Eeeu0xXdbba9frFj0=OqFfea0dXdd9vqai=hGuQ8kuc9pgc9s8qqaq=dirpe0xb9q8qiLsFr0=vr0=vr0dc8meaabaqaciaacaGaaeqabaqabeGadaaakeaaiiGacqWFdpWCdaqhaaWcbaGae83UdWgabaGaeyOeI0IaeGOmaidaaaaa@3231@*I*_*K'*_.

#### Normal prior with the covariance parameter depending on Ψ

Let us denote each column of the Λ matrix with Λ^*k *^where *k *= 1,..., *K*. A convenient conjugate prior for Λ^*k *^is the Gaussian distribution. Utsugi and Kumagai [14] set the mean of this distribution to zero and the covariance matrix to α2−1
 MathType@MTEF@5@5@+=feaafiart1ev1aaatCvAUfKttLearuWrP9MDH5MBPbIqV92AaeXatLxBI9gBaebbnrfifHhDYfgasaacH8akY=wiFfYdH8Gipec8Eeeu0xXdbba9frFj0=OqFfea0dXdd9vqai=hGuQ8kuc9pgc9s8qqaq=dirpe0xb9q8qiLsFr0=vr0=vr0dc8meaabaqaciaacaGaaeqabaqabeGadaaakeaaiiGacqWFXoqydaqhaaWcbaGaeGOmaidabaGaeyOeI0IaeGymaedaaaaa@314E@Ψ. That is,

Λ^*k *^~ N
 MathType@MTEF@5@5@+=feaafiart1ev1aaatCvAUfKttLearuWrP9MDH5MBPbIqV92AaeXatLxBI9gBaebbnrfifHhDYfgasaacH8akY=wiFfYdH8Gipec8Eeeu0xXdbba9frFj0=OqFfea0dXdd9vqai=hGuQ8kuc9pgc9s8qqaq=dirpe0xb9q8qiLsFr0=vr0=vr0dc8meaabaqaciaacaGaaeqabaqabeGadaaakeaat0uy0HwzTfgDPnwy1egaryqtHrhAL1wy0L2yHvdaiqaacqWFneVtaaa@383A@(0, α2−1
 MathType@MTEF@5@5@+=feaafiart1ev1aaatCvAUfKttLearuWrP9MDH5MBPbIqV92AaeXatLxBI9gBaebbnrfifHhDYfgasaacH8akY=wiFfYdH8Gipec8Eeeu0xXdbba9frFj0=OqFfea0dXdd9vqai=hGuQ8kuc9pgc9s8qqaq=dirpe0xb9q8qiLsFr0=vr0=vr0dc8meaabaqaciaacaGaaeqabaqabeGadaaakeaaiiGacqWFXoqydaqhaaWcbaGaeGOmaidabaGaeyOeI0IaeGymaedaaaaa@314E@ Ψ)

where Ψ is the covariance of the noise. Thus, if the data are noisy then the above prior assigns large magnitude to the vector Λ^*k*^, while free of noise data suggest small magnitudes for Λ^*k*^.

The posterior distribution of the combined matrix Λ¯
 MathType@MTEF@5@5@+=feaafiart1ev1aaatCvAUfKttLearuWrP9MDH5MBPbIqV92AaeXatLxBI9gBaebbnrfifHhDYfgasaacH8akY=wiFfYdH8Gipec8Eeeu0xXdbba9frFj0=OqFfea0dXdd9vqai=hGuQ8kuc9pgc9s8qqaq=dirpe0xb9q8qiLsFr0=vr0=vr0dc8meaabaqaciaacaGaaeqabaqabeGadaaakeaacuqHBoatgaqeaaaa@2E39@ = [*μ*, Λ] (see equation 6 for the prior on *μ*) is given

by

Λ¯|X,F,Ψ,α1,α2mΛ¯∗ΣΛ¯∗  ~N(mΛ¯∗,ΣΛ¯∗)=CXF(CFF+A)−1=(CFF+A)−1⊗Ψ
 MathType@MTEF@5@5@+=feaafiart1ev1aaatCvAUfKttLearuWrP9MDH5MBPbIqV92AaeXatLxBI9gBaebbnrfifHhDYfgasaacH8akY=wiFfYdH8Gipec8Eeeu0xXdbba9frFj0=OqFfea0dXdd9vqai=hGuQ8kuc9pgc9s8qqaq=dirpe0xb9q8qiLsFr0=vr0=vr0dc8meaabaqaciaacaGaaeqabaqabeGadaaakeaafaqaceWabaaabaGafu4MdWKbaebacqqG8baFcqWGybawcqqGSaalcqWGgbGrcqqGSaalcqqHOoqwcqqGSaaliiGacqWFXoqydaWgaaWcbaGaeeymaedabeaakiabcYcaSiab=f7aHnaaBaaaleaacqaIYaGmaeqaaaGcbaGaemyBa02aa0baaSqaaiqbfU5amzaaraaabaGaey4fIOcaaaGcbaGaeu4Odm1aa0baaSqaaiqbfU5amzaaraaabaGaey4fIOcaaaaakiabbccaGiabbccaGuaabaqadiaaaeaacqGG+bGFaeaat0uy0HwzTfgDPnwy1egaryqtHrhAL1wy0L2yHvdaiqaacqGFneVtcqGGOaakcqWGTbqBdaqhaaWcbaGafu4MdWKbaebaaeaacqGHxiIkaaGccqGGSaalcqqHJoWudaqhaaWcbaGafu4MdWKbaebaaeaacqGHxiIkaaGccqGGPaqkaeaacqGH9aqpaeaacqWGdbWqdaWgaaWcbaGaemiwaGLaemOrayeabeaakiabcIcaOiabdoeadnaaBaaaleaacqWGgbGrcqWGgbGraeqaaOGaey4kaSIaemyqaeKaeiykaKYaaWbaaSqabeaacqGHsislcqaIXaqmaaaakeaacqGH9aqpaeaacqGGOaakcqWGdbWqdaWgaaWcbaGaemOrayKaemOrayeabeaakiabgUcaRiabdgeabjabcMcaPmaaCaaaleqabaGaeyOeI0IaeGymaedaaOGaey4LIqSaeuiQdKfaaaaa@7A01@

where ⊗ is the Kronecker tensor product,

f¯=[1,(fn)']'A=diag(α1,α2IK)CXF=∑n=1Nxn(f¯n)'CFF=∑n=1Nf¯n(f¯n)'
 MathType@MTEF@5@5@+=feaafiart1ev1aaatCvAUfKttLearuWrP9MDH5MBPbIqV92AaeXatLxBI9gBaebbnrfifHhDYfgasaacH8akY=wiFfYdH8Gipec8Eeeu0xXdbba9frFj0=OqFfea0dXdd9vqai=hGuQ8kuc9pgc9s8qqaq=dirpe0xb9q8qiLsFr0=vr0=vr0dc8meaabaqaciaacaGaaeqabaqabeGadaaakeaafaqadeabdaaaaeaacuWGMbGzgaqeaaqaaiabg2da9aqaaiabcUfaBjabigdaXiabcYcaSiabcIcaOiabdAgaMnaaCaaaleqabaGaemOBa4gaaOGaeiykaKYaaWbaaSqabeaatCvAUfeBSjuyZL2yd9gzLbvyNv2CaeHbuLwBLnhiov2DGi1BTfMBaGabciaa=DcaaaGccqGGDbqxdaahaaWcbeqaaiaa=DcaaaaakeaacqWGbbqqaeaacqGH9aqpaeaaieaacqGFKbazcqGFPbqAcqGFHbqycqGFNbWzcqGGOaakiiGacqqFXoqydaWgaaWcbaGaeGymaedabeaakiabcYcaSiab9f7aHnaaBaaaleaacqaIYaGmaeqaaOGaemysaK0aaSbaaSqaaiabdUealbqabaGccqGGPaqkaeaacqWGdbWqdaWgaaWcbaGaemiwaGLaemOrayeabeaaaOqaaiabg2da9aqaamaaqahabaGaemiEaG3aaWbaaSqabeaacqWGUbGBaaGccqGGOaakcuWGMbGzgaqeamaaCaaaleqabaGaemOBa4gaaOGaeiykaKYaaWbaaSqabeaacaWFNaaaaaqaaiabd6gaUjabg2da9iabigdaXaqaaiabd6eaobqdcqGHris5aaGcbaGaem4qam0aaSbaaSqaaiabdAeagjabdAeagbqabaaakeaacqGH9aqpaeaadaaeWbqaaiqbdAgaMzaaraWaaWbaaSqabeaacqWGUbGBaaGccqGGOaakcuWGMbGzgaqeamaaCaaaleqabaGaemOBa4gaaOGaeiykaKYaaWbaaSqabeaacaWFNaaaaaqaaiabd6gaUjabg2da9iabigdaXaqaaiabd6eaobqdcqGHris5aaaaaaa@80C6@

and *I*_*K *_is a *K *dimensional vector of ones.

Moreover, Utsugi and Kumagai [14] suggest the use of a Gamma hyperprior on the parameters *α*_1 _and *α*_2_. That is,

α1|αα1,βα1~G(αα1,βα1)α2|αα2,βα2~G(αα2,βα2)
 MathType@MTEF@5@5@+=feaafiart1ev1aaatCvAUfKttLearuWrP9MDH5MBPbIqV92AaeXatLxBI9gBaebbnrfifHhDYfgasaacH8akY=wiFfYdH8Gipec8Eeeu0xXdbba9frFj0=OqFfea0dXdd9vqai=hGuQ8kuc9pgc9s8qqaq=dirpe0xb9q8qiLsFr0=vr0=vr0dc8meaabaqaciaacaGaaeqabaqabeGadaaakeaafaqabeGabaaabaacciGae8xSde2aaSbaaSqaaiabigdaXaqabaGccqGG8baFcqWFXoqydaWgaaWcbaGae8xSde2aaSbaaWqaaiabigdaXaqabaaaleqaaOGaeiilaWIae8NSdi2aaSbaaSqaaiab=f7aHnaaBaaameaacqaIXaqmaeqaaaWcbeaakiabc6ha+nrtHrhAL1wy0L2yHvtyaeHbnfgDOvwBHrxAJfwnaGabaiab+zq8hjabcIcaOiab=f7aHnaaBaaaleaacqWFXoqydaWgaaadbaGaeGymaedabeaaaSqabaGccqGGSaalcqWFYoGydaWgaaWcbaGae8xSde2aaSbaaWqaaiabigdaXaqabaaaleqaaOGaeiykaKcabaGae8xSde2aaSbaaSqaaiabikdaYaqabaGccqGG8baFcqWFXoqydaWgaaWcbaGae8xSde2aaSbaaWqaaiabikdaYaqabaaaleqaaOGaeiilaWIae8NSdi2aaSbaaSqaaiab=f7aHnaaBaaameaacqaIYaGmaeqaaaWcbeaakiabc6ha+jab+zq8hjabcIcaOiab=f7aHnaaBaaaleaacqWFXoqydaWgaaadbaGaeGOmaidabeaaliabcYcaSaqabaGccqWFYoGydaWgaaWcbaGae8xSde2aaSbaaWqaaiabikdaYaqabaaaleqaaOGaeiykaKcaaaaa@7114@

The posterior distributions of those hyperparameters are also Gamma distributions given by

α1|X,μ,Ψ,αα1,βα1~G(12P+αα1,12μ′Ψ−1μ+βα1)α2|X,Λ,Ψ,αα2,βα2~G(12PK+αα2,12tr(Λ′Ψ−1Λ)+βα2)
 MathType@MTEF@5@5@+=feaafiart1ev1aaatCvAUfKttLearuWrP9MDH5MBPbIqV92AaeXatLxBI9gBaebbnrfifHhDYfgasaacH8akY=wiFfYdH8Gipec8Eeeu0xXdbba9frFj0=OqFfea0dXdd9vqai=hGuQ8kuc9pgc9s8qqaq=dirpe0xb9q8qiLsFr0=vr0=vr0dc8meaabaqaciaacaGaaeqabaqabeGadaaakeaafaqabeGabaaabaacciGae8xSde2aaSbaaSqaaiabigdaXaqabaGccqGG8baFcqWGybawcqGGSaalcqWF8oqBcqGGSaalcqqHOoqwcqGGSaalcqWFXoqydaWgaaWcbaGae8xSde2aaSbaaWqaaiabigdaXaqabaaaleqaaOGaeiilaWIae8NSdi2aaSbaaSqaaiab=f7aHnaaBaaameaacqaIXaqmaeqaaaWcbeaakiabc6ha+nrtHrhAL1wy0L2yHvtyaeHbnfgDOvwBHrxAJfwnaGabaiab+zq8hnaabmaabaWaaSaaaeaacqaIXaqmaeaacqaIYaGmaaGaemiuaaLaey4kaSIae8xSde2aaSbaaSqaaiab=f7aHnaaBaaameaacqaIXaqmaeqaaaWcbeaakiabcYcaSmaalaaabaGaeGymaedabaGaeGOmaidaaiqb=X7aTzaafaGaeuiQdK1aaWbaaSqabeaacqGHsislcqaIXaqmaaGccqWF8oqBcqGHRaWkcqWFYoGydaWgaaWcbaGae8xSde2aaSbaaWqaaiabigdaXaqabaaaleqaaaGccaGLOaGaayzkaaaabaGae8xSde2aaSbaaSqaaiabikdaYaqabaGccqGG8baFcqWGybawcqGGSaalcqqHBoatcqGGSaalcqqHOoqwcqGGSaalcqWFXoqydaWgaaWcbaGae8xSde2aaSbaaWqaaiabikdaYaqabaaaleqaaOGaeiilaWIae8NSdi2aaSbaaSqaaiab=f7aHnaaBaaameaacqaIYaGmaeqaaaWcbeaakiabc6ha+jab+zq8hnaabmaabaWaaSaaaeaacqaIXaqmaeaacqaIYaGmaaGaemiuaaLaem4saSKaey4kaSIae8xSde2aaSbaaSqaaiab=f7aHnaaBaaameaacqaIYaGmaeqaaaWcbeaakiabcYcaSmaalaaabaGaeGymaedabaGaeGOmaidaaiabdsha0jabdkhaYjabcIcaOiqbfU5amzaafaGaeuiQdK1aaWbaaSqabeaacqGHsislcqaIXaqmaaGccqqHBoatcqGGPaqkcqGHRaWkcqWFYoGydaWgaaWcbaGae8xSde2aaSbaaWqaaiabikdaYaqabaaaleqaaaGccaGLOaGaayzkaaaaaaaa@9FB3@

### Noise covariance matrix Ψ

A convenient conjugate prior is assigned to the inverse of the noise covariance matrix Ψ so that its posterior distribution has a known form. Thus, the prior on each ψp−2
 MathType@MTEF@5@5@+=feaafiart1ev1aaatCvAUfKttLearuWrP9MDH5MBPbIqV92AaeXatLxBI9gBaebbnrfifHhDYfgasaacH8akY=wiFfYdH8Gipec8Eeeu0xXdbba9frFj0=OqFfea0dXdd9vqai=hGuQ8kuc9pgc9s8qqaq=dirpe0xb9q8qiLsFr0=vr0=vr0dc8meaabaqaciaacaGaaeqabaqabeGadaaakeaaiiGacqWFipqEdaqhaaWcbaGaemiCaahabaGaeyOeI0IaeGOmaidaaaaa@31F6@ is a Gamma distribution given by

p(ψp−2|αΨ,βΨ)=G(ψp−2|αΨ,βΨ)∝(ψp−2)αΨ−1exp⁡(−ψp−2βΨ)
 MathType@MTEF@5@5@+=feaafiart1ev1aaatCvAUfKttLearuWrP9MDH5MBPbIqV92AaeXatLxBI9gBaebbnrfifHhDYfgasaacH8akY=wiFfYdH8Gipec8Eeeu0xXdbba9frFj0=OqFfea0dXdd9vqai=hGuQ8kuc9pgc9s8qqaq=dirpe0xb9q8qiLsFr0=vr0=vr0dc8meaabaqaciaacaGaaeqabaqabeGadaaakeaacqWGWbaCcqGGOaakiiGacqWFipqEdaqhaaWcbaGaemiCaahabaGaeyOeI0IaeGOmaidaaOGaeiiFaWNae8xSde2aaSbaaSqaaiabfI6azbqabaGccqGGSaalcqWFYoGydaWgaaWcbaGaeuiQdKfabeaakiabcMcaPiabg2da9mrtHrhAL1wy0L2yHvtyaeHbnfgDOvwBHrxAJfwnaGabaiab+zq8hjabcIcaOiab=H8a5naaDaaaleaacqWGWbaCaeaacqGHsislcqaIYaGmaaGccqGG8baFcqWFXoqydaWgaaWcbaGaeuiQdKfabeaakiabcYcaSiab=j7aInaaBaaaleaacqqHOoqwaeqaaOGaeiykaKIaeyyhIuRaeiikaGIae8hYdK3aa0baaSqaaiabdchaWbqaaiabgkHiTiabikdaYaaakiabcMcaPmaaCaaaleqabaGae8xSde2aaSbaaWqaaiabfI6azbqabaWccqGHsislcqaIXaqmaaGccyGGLbqzcqGG4baEcqGGWbaCcqGGOaakcqGHsislcqWFipqEdaqhaaWcbaGaemiCaahabaGaeyOeI0IaeGOmaidaaOGae8NSdi2aaSbaaSqaaiabfI6azbqabaGccqGGPaqkaaa@7845@

The Gamma posterior distribution of *ψ*^-2 ^in West [3], Sabatti and James [4], and Fokoue [16] is given by

p(ψp−2|X,F,Λ,μ)∝p(ψp−2|αΨ,βΨ)p(X|F,Λ,μ,Ψ)=G(ψp−2|αΨ+12P,βΨ+12Spp)
 MathType@MTEF@5@5@+=feaafiart1ev1aaatCvAUfKttLearuWrP9MDH5MBPbIqV92AaeXatLxBI9gBaebbnrfifHhDYfgasaacH8akY=wiFfYdH8Gipec8Eeeu0xXdbba9frFj0=OqFfea0dXdd9vqai=hGuQ8kuc9pgc9s8qqaq=dirpe0xb9q8qiLsFr0=vr0=vr0dc8meaabaqaciaacaGaaeqabaqabeGadaaakeaacqWGWbaCcqGGOaakiiGacqWFipqEdaqhaaWcbaGaemiCaahabaGaeyOeI0IaeGOmaidaaOGaeiiFaWNaemiwaGLaeiilaWIaemOrayKaeiilaWIaeu4MdWKaeiilaWIae8hVd0MaeiykaKIaeyyhIuRaemiCaaNaeiikaGIae8hYdK3aa0baaSqaaiabdchaWbqaaiabgkHiTiabikdaYaaakiabcYha8jab=f7aHnaaBaaaleaacqqHOoqwaeqaaOGaeiilaWIae8NSdi2aaSbaaSqaaiabfI6azbqabaGccqGGPaqkcqWGWbaCcqGGOaakcqWGybawcqGG8baFcqWGgbGrcqGGSaalcqqHBoatcqGGSaalcqWF8oqBcqGGSaalcqqHOoqwcqGGPaqkcqGH9aqpt0uy0HwzTfgDPnwy1egaryqtHrhAL1wy0L2yHvdaiqaacqGFge=rdaqadaqaaiab=H8a5naaDaaaleaacqWGWbaCaeaacqGHsislcqaIYaGmaaGccqGG8baFcqWFXoqydaWgaaWcbaGaeuiQdKfabeaakiabgUcaRmaalaaabaGaeGymaedabaGaeGOmaidaaiabdcfaqjabcYcaSiab=j7aInaaBaaaleaacqqHOoqwaeqaaOGaey4kaSYaaSaaaeaacqaIXaqmaeaacqaIYaGmaaGaem4uam1aaSbaaSqaaiabdchaWjabdchaWbqabaaakiaawIcacaGLPaaaaaa@876E@

where

Spp=∑n=1N∑p=1P(xpn−μp−∑k=1Kλpkfkn)2
 MathType@MTEF@5@5@+=feaafiart1ev1aaatCvAUfKttLearuWrP9MDH5MBPbIqV92AaeXatLxBI9gBaebbnrfifHhDYfgasaacH8akY=wiFfYdH8Gipec8Eeeu0xXdbba9frFj0=OqFfea0dXdd9vqai=hGuQ8kuc9pgc9s8qqaq=dirpe0xb9q8qiLsFr0=vr0=vr0dc8meaabaqaciaacaGaaeqabaqabeGadaaakeaacqWGtbWudaWgaaWcbaGaemiCaaNaemiCaahabeaakiabg2da9maaqahabaWaaabCaeaacqGGOaakcqWG4baEdaqhaaWcbaGaemiCaahabaGaemOBa4gaaOGaeyOeI0ccciGae8hVd02aaSbaaSqaaiabdchaWbqabaGccqGHsisldaaeWbqaaiab=T7aSnaaBaaaleaacqWGWbaCcqWGRbWAaeqaaOGaemOzay2aa0baaSqaaiabdUgaRbqaaiabd6gaUbaaaeaacqWGRbWAcqGH9aqpcqaIXaqmaeaacqWGlbWsa0GaeyyeIuoakiabcMcaPmaaCaaaleqabaGaeGOmaidaaaqaaiabdchaWjabg2da9iabigdaXaqaaiabdcfaqbqdcqGHris5aaWcbaGaemOBa4Maeyypa0JaeGymaedabaGaemOta4eaniabggHiLdaaaa@5B97@

While the Gamma posterior distribution of *ψ*^-2 ^in Utsugi and Kumagai [14] has a more complicated form since Ψ is tied to the covariance matrices of both *μ*, and Λ. Thus, it has the following form

p(Ψ−1|X,F,Λ¯,α1,α2)∝p(Ψ−1|αΨ,βΨ)p(X|F,Λ,μ,Ψ,α1,α2)=G(Ψ−1|αΨ∗,βΨ∗)
 MathType@MTEF@5@5@+=feaafiart1ev1aaatCvAUfKttLearuWrP9MDH5MBPbIqV92AaeXatLxBI9gBaebbnrfifHhDYfgasaacH8akY=wiFfYdH8Gipec8Eeeu0xXdbba9frFj0=OqFfea0dXdd9vqai=hGuQ8kuc9pgc9s8qqaq=dirpe0xb9q8qiLsFr0=vr0=vr0dc8meaabaqaciaacaGaaeqabaqabeGadaaakeaacqWGWbaCcqGGOaakcqqHOoqwdaahaaWcbeqaaiabgkHiTiabigdaXaaakiabcYha8jabdIfayjabcYcaSiabdAeagjabcYcaSiqbfU5amzaaraGaeiilaWccciGae8xSde2aaSbaaSqaaiabigdaXaqabaGccqGGSaalcqWFXoqydaWgaaWcbaGaeGOmaidabeaakiabcMcaPiabg2Hi1kabdchaWjabcIcaOiabfI6aznaaCaaaleqabaGaeyOeI0IaeGymaedaaOGaeiiFaWNae8xSde2aaSbaaSqaaiabfI6azbqabaGccqGGSaalcqWFYoGydaWgaaWcbaGaeuiQdKfabeaakiabcMcaPiabdchaWjabcIcaOiabdIfayjabcYha8jabdAeagjabcYcaSiabfU5amjabcYcaSiab=X7aTjabcYcaSiabfI6azjabcYcaSiab=f7aHnaaBaaaleaacqaIXaqmaeqaaOGaeiilaWIae8xSde2aaSbaaSqaaiabikdaYaqabaGccqGGPaqkcqGH9aqpt0uy0HwzTfgDPnwy1egaryqtHrhAL1wy0L2yHvdaiqaacqGFge=rcqGGOaakcqqHOoqwdaahaaWcbeqaaiabgkHiTiabigdaXaaakiabcYha8jab=f7aHnaaDaaaleaacqqHOoqwaeaacqGHxiIkaaGccqGGSaalcqWFYoGydaqhaaWcbaGaeuiQdKfabaGaey4fIOcaaOGaeiykaKcaaa@858B@

where

αΨ*=N+K+2αΨ+12βΨ*=12diag(CXX−2CXFΛ¯′+Λ¯(CFF+A)Λ¯′+2βΨIP)
 MathType@MTEF@5@5@+=feaafiart1ev1aaatCvAUfKttLearuWrP9MDH5MBPbIqV92AaeXatLxBI9gBaebbnrfifHhDYfgasaacH8akY=wiFfYdH8Gipec8Eeeu0xXdbba9frFj0=OqFfea0dXdd9vqai=hGuQ8kuc9pgc9s8qqaq=dirpe0xb9q8qiLsFr0=vr0=vr0dc8meaabaqaciaacaGaaeqabaqabeGadaaakqaabeqaaGGaciab=f7aHnaaDaaaleaacqqHOoqwaeaacqGGQaGkaaGccqGH9aqpdaWcaaqaaiabd6eaojabgUcaRiabdUealjabgUcaRiabikdaYiab=f7aHnaaBaaaleaacqqHOoqwaeqaaOGaey4kaSIaeGymaedabaGaeGOmaidaaaqaaiab=j7aInaaDaaaleaacqqHOoqwaeaacqGGQaGkaaGccqGH9aqpdaWcaaqaaiabigdaXaqaaiabikdaYaaaieaacqGFKbazcqGFPbqAcqGFHbqycqGFNbWzcqGGOaakcqWGdbWqdaWgaaWcbaGaemiwaGLaemiwaGfabeaakiabgkHiTiabikdaYiabdoeadnaaBaaaleaacqWGybawcqWGgbGraeqaaOGafu4MdWKbaeHbauaacqGHRaWkcuqHBoatgaqeaiabcIcaOiabdoeadnaaBaaaleaacqWGgbGrcqWGgbGraeqaaOGaey4kaSIaemyqaeKaeiykaKIafu4MdWKbaeHbauaacqGHRaWkcqaIYaGmcqWFYoGydaWgaaWcbaGaeuiQdKfabeaakiabdMeajnaaBaaaleaacqWGqbauaeqaaOGaeiykaKcaaaa@68DE@

where *I*_*p *_is the identity matrix of *P *× *P *dimensions and

CXX=∑n=1Nxn(xn)′
 MathType@MTEF@5@5@+=feaafiart1ev1aaatCvAUfKttLearuWrP9MDH5MBPbIqV92AaeXatLxBI9gBaebbnrfifHhDYfgasaacH8akY=wiFfYdH8Gipec8Eeeu0xXdbba9frFj0=OqFfea0dXdd9vqai=hGuQ8kuc9pgc9s8qqaq=dirpe0xb9q8qiLsFr0=vr0=vr0dc8meaabaqaciaacaGaaeqabaqabeGadaaakeaacqWGdbWqdaWgaaWcbaGaemiwaGLaemiwaGfabeaakiabg2da9maaqahabaGaemiEaG3aaWbaaSqabeaacqWGUbGBaaGccqGGOaakcqWG4baEdaahaaWcbeqaaiabd6gaUbaakiqbcMcaPyaafaaaleaacqWGUbGBcqGH9aqpcqaIXaqmaeaacqWGobGta0GaeyyeIuoaaaa@4013@

West [3], and Sabatti and James [4] use a common variance *ψ*^-2 ^for all dimensions *P*, while Ghahramani and Hinton [13], Utsugi and Kumagai [14], and Fokoue [16] allow the model to estimate a different variance ψp−2
 MathType@MTEF@5@5@+=feaafiart1ev1aaatCvAUfKttLearuWrP9MDH5MBPbIqV92AaeXatLxBI9gBaebbnrfifHhDYfgasaacH8akY=wiFfYdH8Gipec8Eeeu0xXdbba9frFj0=OqFfea0dXdd9vqai=hGuQ8kuc9pgc9s8qqaq=dirpe0xb9q8qiLsFr0=vr0=vr0dc8meaabaqaciaacaGaaeqabaqabeGadaaakeaaiiGacqWFipqEdaqhaaWcbaGaemiCaahabaGaeyOeI0IaeGOmaidaaaaa@31F6@ in each dimension *p*. We also suggest the use of a second level of hyperpriors on the scale parameter of *ψ*^-2 ^since it gives a greater flexibility to the model. The cost of this greater flexibility is that more parameters have to be estimated, but this disadvantage is compensated for by the easier assignment of the hyperparameters and the better estimation of the noise covariance matrix. We assign a Gamma prior on *β*_Ψ _with parameters αβΨ
 MathType@MTEF@5@5@+=feaafiart1ev1aaatCvAUfKttLearuWrP9MDH5MBPbIqV92AaeXatLxBI9gBaebbnrfifHhDYfgasaacH8akY=wiFfYdH8Gipec8Eeeu0xXdbba9frFj0=OqFfea0dXdd9vqai=hGuQ8kuc9pgc9s8qqaq=dirpe0xb9q8qiLsFr0=vr0=vr0dc8meaabaqaciaacaGaaeqabaqabeGadaaakeaaiiGacqWFXoqydaWgaaWcbaGae8NSdi2aaSbaaWqaaiabfI6azbqabaaaleqaaaaa@31E1@ and 1/ββΨ
 MathType@MTEF@5@5@+=feaafiart1ev1aaatCvAUfKttLearuWrP9MDH5MBPbIqV92AaeXatLxBI9gBaebbnrfifHhDYfgasaacH8akY=wiFfYdH8Gipec8Eeeu0xXdbba9frFj0=OqFfea0dXdd9vqai=hGuQ8kuc9pgc9s8qqaq=dirpe0xb9q8qiLsFr0=vr0=vr0dc8meaabaqaciaacaGaaeqabaqabeGadaaakeaacqaIXaqmcqGGVaWliiGacqWFYoGydaWgaaWcbaGae8NSdi2aaSbaaWqaaiabfI6azbqabaaaleqaaaaa@33B9@. The posterior distribution is given by

p(βΨ|X,Ψ)∝p(βΨ|αβΨ,ββΨ)p(ψp−2|αΨ,βΨ)=G(βΨ|αβΨ+αΨP,ββΨ+tr(Ψ−1))
 MathType@MTEF@5@5@+=feaafiart1ev1aaatCvAUfKttLearuWrP9MDH5MBPbIqV92AaeXatLxBI9gBaebbnrfifHhDYfgasaacH8akY=wiFfYdH8Gipec8Eeeu0xXdbba9frFj0=OqFfea0dXdd9vqai=hGuQ8kuc9pgc9s8qqaq=dirpe0xb9q8qiLsFr0=vr0=vr0dc8meaabaqaciaacaGaaeqabaqabeGadaaakeaacqWGWbaCcqGGOaakiiGacqWFYoGydaWgaaWcbaGaeuiQdKfabeaakiabcYha8jabdIfayjabcYcaSiabfI6azjabcMcaPiabg2Hi1kabdchaWjabcIcaOiab=j7aInaaBaaaleaacqqHOoqwaeqaaOGaeiiFaWNae8xSde2aaSbaaSqaaiab=j7aInaaBaaameaacqqHOoqwaeqaaaWcbeaakiabcYcaSiab=j7aInaaBaaaleaacqWFYoGydaWgaaadbaGaeuiQdKfabeaaaSqabaGccqGGPaqkcqWGWbaCcqGGOaakcqWFipqEdaqhaaWcbaGaemiCaahabaGaeyOeI0IaeGOmaidaaOGaeiiFaWNae8xSde2aaSbaaSqaaiabfI6azbqabaGccqGGSaalcqWFYoGydaWgaaWcbaGaeuiQdKfabeaakiabcMcaPiabg2da9mrtHrhAL1wy0L2yHvtyaeHbnfgDOvwBHrxAJfwnaGabaiab+zq8hjabcIcaOiab=j7aInaaBaaaleaacqqHOoqwaeqaaOGaeiiFaWNae8xSde2aaSbaaSqaaiab=j7aInaaBaaameaacqqHOoqwaeqaaaWcbeaakiabgUcaRiab=f7aHnaaBaaaleaacqqHOoqwaeqaaOGaemiuaaLaeiilaWIae8NSdi2aaSbaaSqaaiab=j7aInaaBaaameaacqqHOoqwaeqaaaWcbeaakiabgUcaRiabdsha0jabdkhaYjabcIcaOiabfI6aznaaCaaaleqabaGaeyOeI0IaeGymaedaaOGaeiykaKIaeiykaKcaaa@8B82@

### Rotation of Λ matrix

We are usually interested in those rotations that result in interpretable factor loadings matrix. For example, a matrix that has as few nonzero loadings as possible. In a biological context that means that each gene is regulated by a small number of TFs. The algorithms of West [3] and Fokoue [16] implicitly look for sparse matrices. However, this is not true for the classical FA algorithm and the algorithms of Ghahramani and Hinton [13], and Utsugi and Kumagai [14]. As shown in the results section, the performance of these algorithms can be improved by applying an additional orthogonal rotation *Q *on the learned factor loadings matrix that leads to a sparse one Λ_*rot*_, Λ_*rot *_= Λ*Q*.

Since different orthogonal rotation methods have different constraints as we discuss next, they can lead to different factor loadings matrix. Thus, a unique solution can not be achieved if a prior information regarding the position of the zeros in the factor loadings matrix is not given.

A number of metrics can be used as a measure of sparsity. For example, the *varimax *rotation [21] maximizes the row variances of the squares of the loadings.

∑k=1K(∑p=1Pλpk4−(∑p=1Pλpk2)2)
 MathType@MTEF@5@5@+=feaafiart1ev1aaatCvAUfKttLearuWrP9MDH5MBPbIqV92AaeXatLxBI9gBaebbnrfifHhDYfgasaacH8akY=wiFfYdH8Gipec8Eeeu0xXdbba9frFj0=OqFfea0dXdd9vqai=hGuQ8kuc9pgc9s8qqaq=dirpe0xb9q8qiLsFr0=vr0=vr0dc8meaabaqaciaacaGaaeqabaqabeGadaaakeaadaaeWbqaamaabmaabaWaaabCaeaaiiGacqWF7oaBdaqhaaWcbaGaemiCaaNaem4AaSgabaGaeGinaqdaaaqaaiabdchaWjabg2da9iabigdaXaqaaiabdcfaqbqdcqGHris5aOGaeyOeI0IaeiikaGYaaabCaeaacqWF7oaBdaqhaaWcbaGaemiCaaNaem4AaSgabaGaeGOmaidaaaqaaiabdchaWjabg2da9iabigdaXaqaaiabdcfaqbqdcqGHris5aOGaeiykaKYaaWbaaSqabeaacqaIYaGmaaaakiaawIcacaGLPaaaaSqaaiabdUgaRjabg2da9iabigdaXaqaaiabdUealbqdcqGHris5aaaa@5181@

Similarly, the *quartimax *rotation maximizes the column variances of the squares of the loadings (using that the sum of squares along columns is constant).

∑p=1P∑k=1Kλpk4
 MathType@MTEF@5@5@+=feaafiart1ev1aaatCvAUfKttLearuWrP9MDH5MBPbIqV92AaeXatLxBI9gBaebbnrfifHhDYfgasaacH8akY=wiFfYdH8Gipec8Eeeu0xXdbba9frFj0=OqFfea0dXdd9vqai=hGuQ8kuc9pgc9s8qqaq=dirpe0xb9q8qiLsFr0=vr0=vr0dc8meaabaqaciaacaGaaeqabaqabeGadaaakeaadaaeWbqaamaaqahabaacciGae83UdW2aa0baaSqaaiabdchaWjabdUgaRbqaaiabisda0aaaaeaacqWGRbWAcqGH9aqpcqaIXaqmaeaacqWGlbWsa0GaeyyeIuoaaSqaaiabdchaWjabg2da9iabigdaXaqaaiabdcfaqbqdcqGHris5aaaa@3FC7@

The *equamax *rotation is something between the varimax and quartimax rotation and gives better results for dense matrices.

∑k=1K(∑p=1Pλpk4−K2(∑p=1Pλpk2)2)
 MathType@MTEF@5@5@+=feaafiart1ev1aaatCvAUfKttLearuWrP9MDH5MBPbIqV92AaeXatLxBI9gBaebbnrfifHhDYfgasaacH8akY=wiFfYdH8Gipec8Eeeu0xXdbba9frFj0=OqFfea0dXdd9vqai=hGuQ8kuc9pgc9s8qqaq=dirpe0xb9q8qiLsFr0=vr0=vr0dc8meaabaqaciaacaGaaeqabaqabeGadaaakeaadaaeWbqaamaabmaabaWaaabCaeaaiiGacqWF7oaBdaqhaaWcbaGaemiCaaNaem4AaSgabaGaeGinaqdaaaqaaiabdchaWjabg2da9iabigdaXaqaaiabdcfaqbqdcqGHris5aOGaeyOeI0YaaSaaaeaacqWGlbWsaeaacqaIYaGmaaGaeiikaGYaaabCaeaacqWF7oaBdaqhaaWcbaGaemiCaaNaem4AaSgabaGaeGOmaidaaaqaaiabdchaWjabg2da9iabigdaXaqaaiabdcfaqbqdcqGHris5aOGaeiykaKYaaWbaaSqabeaacqaIYaGmaaaakiaawIcacaGLPaaaaSqaaiabdUgaRjabg2da9iabigdaXaqaaiabdUealbqdcqGHris5aaaa@53A2@

We suggest a new method, the tanh rotation. It penalizes small deviations from zero but keeps the penalty constant for values far from zero.

∑k=1K∑p=1Ptanh⁡(αλpk2)
 MathType@MTEF@5@5@+=feaafiart1ev1aaatCvAUfKttLearuWrP9MDH5MBPbIqV92AaeXatLxBI9gBaebbnrfifHhDYfgasaacH8akY=wiFfYdH8Gipec8Eeeu0xXdbba9frFj0=OqFfea0dXdd9vqai=hGuQ8kuc9pgc9s8qqaq=dirpe0xb9q8qiLsFr0=vr0=vr0dc8meaabaqaciaacaGaaeqabaqabeGadaaakeaadaaeWbqaamaaqahabaGagiiDaqNaeiyyaeMaeiOBa4MaeiiAaGMaeiikaGccciGae8xSdeMae83UdW2aa0baaSqaaiabdchaWjabdUgaRbqaaiabikdaYaaakiabcMcaPaWcbaGaemiCaaNaeyypa0JaeGymaedabaGaemiuaafaniabggHiLdaaleaacqWGRbWAcqGH9aqpcqaIXaqmaeaacqWGlbWsa0GaeyyeIuoaaaa@489C@

where the parameter *α *determines the steepness of the tanh function.

Finally, the *procrustes *rotation [22] results in a factor loadings matrix Λ_*rot *_by minimizing the sum of squared differences to a target matrix *T*,

∑k=1K∑p=1P(λpk−τpk)2
 MathType@MTEF@5@5@+=feaafiart1ev1aaatCvAUfKttLearuWrP9MDH5MBPbIqV92AaeXatLxBI9gBaebbnrfifHhDYfgasaacH8akY=wiFfYdH8Gipec8Eeeu0xXdbba9frFj0=OqFfea0dXdd9vqai=hGuQ8kuc9pgc9s8qqaq=dirpe0xb9q8qiLsFr0=vr0=vr0dc8meaabaqaciaacaGaaeqabaqabeGadaaakeaadaaeWbqaamaaqahabaGaeiikaGccciGae83UdW2aaSbaaSqaaiabdchaWjabdUgaRbqabaGccqGHsislcqWFepaDdaWgaaWcbaGaemiCaaNaem4AaSgabeaakiabcMcaPmaaCaaaleqabaGaeGOmaidaaaqaaiabdchaWjabg2da9iabigdaXaqaaiabdcfaqbqdcqGHris5aaWcbaGaem4AaSMaeyypa0JaeGymaedabaGaem4saSeaniabggHiLdaaaa@4756@

Thus, if the true factor loadings matrix is known, the procrustes method can be used to identify the best possible rotation. However, since this is not usually true for real data, the procrustes method can be used, for example, when assessing FA methods on synthetic data. That is, in this case the target matrix is the true matrix that we try to infer.

## Authors' contributions

Both IP and LW contributed to this paper, and also read and approved the final manuscript.
